# Systematic modulation of charge and spin in graphene nanoribbons on MgO

**DOI:** 10.1038/s41467-025-60767-5

**Published:** 2025-07-01

**Authors:** Amelia Domínguez-Celorrio, Leonard Edens, Sofía Sanz, Manuel Vilas-Varela, Jose Martinez-Castro, Diego Peña, Véronique Langlais, Thomas Frederiksen, José I. Pascual, David Serrate

**Affiliations:** 1 Insituto de Nanociencia y Materiales de Aragón (INMA), CSIC-Universidad de Zaragoza, Zaragoza, E-50009 Spain; 2 School of Physics and Astronomy, Monash University, Clayton, VIC 3800 Australia; 3 ARC Centre for Future Low Energy Electronics Technologies, Monash University, Clayton, VIC 3800 Australia; 4 CIC NanoGUNE BRTA, San Sebastián, E-20018 Spain; 5 Donostia International Physics Center, San Sebastián, E-20018 Spain; 6 Centro Singular de Investigación en Química Bilóxica e Materiais Moleculares (CiQUS) and Departamento de Química Orgánica, Universidade de Santiago de Compostela, Santiago de Compostela, E-15782 Spain; 7 Peter Grünberg Institut (PGI-3), Forschungszentrum Jülich, 52425 Jülich, Germany; 8 Centre d’Elaboration de Materiaux et d’Etudes Structurales, CNRS, Toulouse, F-31055 France; 9 Ikerbasque, Basque Foundation for Science, Bilbao, E-48013 Spain; 10 Departamento de Física de la Materia Condensada, Universidad de Zaragoza, Zaragoza, E-50009 Spain; 11 Laboratorio de Microscopias Avanzadas (LMA), Universidad de Zaragoza, Zaragoza, E-50018 Spain

**Keywords:** Electronic properties and devices, Quantum dots, Magnetic properties and materials, Electronic properties and materials

## Abstract

In order to take full advantage of graphene nanostructures in quantum technologies, their charge and spin state must be precisely controlled. Graphene quantum dots require external gating potentials to tune their ground state. Here, we show systematic manipulation of the electron occupation in graphene nanoribbons lying on MgO layers grown on Ag(001). Owing to the efficient electronic decoupling character of MgO, and the electropositive nature of the substrate, the ribbons host an integer number of electrons that depend on their length and shape. This results in the alternation between a non-magnetic closed-shell state and an open-shell paramagnetic system for even and odd electron occupations respectively. For the odd case, we find a narrow Coulomb correlation gap, which is the smoking gun of its spin-½ state. Comparisons of scanning tunnelling microscopy data with mean-field Hubbard simulations confirm the discretization of the ribbons’ electronic states and charge excess of up to 19 electrons per ribbon.

## Abstract (web summary)

Precise control of charge and spin states in quantum dots is often challenging. Here, the authors show systematic manipulation of the electron occupation in graphene nanoribbons laying on MgO.

## Introduction

Establishing conditions to systematically manipulate the charge state of polycyclic aromatic hydrocarbons is crucial to harness their electrical, chemical and magnetic functionalities. On-surface synthesis (OSS) techniques^1–3^ allow us to manufacture fixed configurations of the conjugated π-electron cloud with designated purposes in nanographenes, but with no control over their post-synthesis electronic occupancy. A paramount example is the effort to trigger the appearance of open-shell magnetic states as a response to the enhanced electronic correlations^4^. Such states are often spatially localized and can be realised by enforcing particular shapes or edge geometries^5–14^, or by defects in the sp^2^ lattice^15,16^. Nanographenes generally lie on a metallic surface after synthesis, which leads to the screening of electron correlations due hybridization effects. On metallic surfaces, small structures can nevertheless display robust intrinsic magnetism due to correlation-induced splitting^5–10,13^. On the contrary, extended structures like graphene nanoribbons (GNR) have shown either no magnetic fingerprints^12,17,18^ or, at most, very small correlation gaps of about ∼10 meV accompanied by a weak spin polarization^19^.

By electrically decoupling the GNRs or other nanographenes from the catalysing metal, we can restore instrinsic electronic correlations and preserve the quantum behaviour associated to their electronic and magnetic degrees of freedom. In addition, the use of insulating spacers is also expected to suppress relaxation channels of electronic and spin quantum states, maintaining the long electron-spin coherence times of free standing carbon-based quantum dots^20,21^. To this end, previous studies explored the intercalation of ultrathin insulators after growing arm-chair GNRs^22,23^. Although the discretization gaps could be measured with much better energy resolution, the electronic decoupling is not enough as to induce any correlation splitting. The end states of arm-chair GNRs have been also assessed on NaCl bilayers on Au(111)^24^ and TiO_2_-rutile^25^, in this case finding correlation gaps at the zig-zag termini very close to the theoretical predictions. Finally, GNRs with longitudinal edges dense in zig-zag segments have been lifted from the Au(111) reactive surface and dropped onto NaCl bilayers^12,26^, finding robust correlation gaps just for the case of pure zig-zag edges^12^. None of these decoupling strategies have provided a way to manipulate the number of singly occupied molecular states. Moreover, the molecular resonances reported so far on decoupling layers exhibit broad linewidths of at least 100 meV, suggesting that these systems are not free from hybridization channels, which is detrimental to applications as spin quantum dots. In fact, it is known that NaCl introduces coupling with phonons^27^ and electronic bands^28,29^.

Inspired by the excellent results of ultra-thin MgO layers as passive support for magnetic atoms and molecules^30–32^, in this work we employ MgO monolayers (MgO_ML_) grown on Ag(001). We show that charge transfer from Ag(001) across the MgO takes place in integer number of electrons, which permits to control the odd/even electron occupation of the quantum-well (QW) states by means of the GNR length. The addition of a single molecular precursor unit (PU) can affect the odd/even occupancy of the GNR. The MgO spacer also enhances electron-electron (e-e) interactions that stabilize odd occupations with correlation gaps of a few tens of meV. This is the signature of a spin-1/2 state associated to odd numbers of electrons in the mean-field-Hubbard (MFH) approximation. The QW states confined at the longitudinal edges of finite GNRs (2 to 12 nm) on MgO_ML_ display an intrinsic linewidth as small as 1 meV.

## Results and discussion

**Fig. 1:**
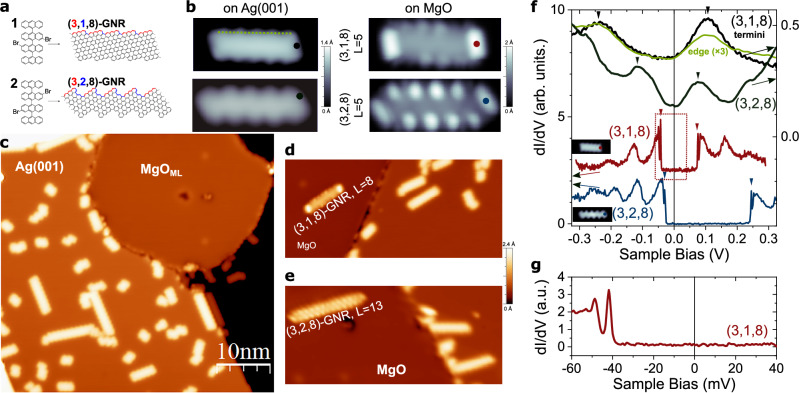
STM characterization of (3,*n*,8)-GNRs on MgO/Ag(001). **a** Chemical structures of the precursors and the resulting (3,1,8)- and (3,2,8)-GNRs. **b** STM topography (*V*_*b*_ = 0.5 V, *I*_*t*_ = 10 - 70 pA, image width 6 nm) of (3,1,8) (upper row) and (3,2,8) (bottom row) GNRs with *L* = 5 precursor units length on Ag(001) and on MgO_ML_/Ag(001). **c** STM image (*V*_*b* _= 0.5 V, *I*_t _= 50 pA) of a MgO_ML_ island coexisting with GNRs synthesized over the regions of bare Ag(001). **d**, **e** Instances of successful atomic manipulation of GNRs from the Ag surface to the MgO island (*V*_b_$=$0.5 V, *I*_t_$=$50 pA). **f** *dI/dV* spectra of the *L* = 5 (3,1,8) and (3,2,8)-GNRs on Ag(001) and on the MgO_ML_ (stabilization*V*_b_$=$0.5 V, *I*_t_$=$100 pA, ${V}_{{\mathrm{mod}}}$ = 1 mV and 8 mV r.m.s. for spectra on MgO and Ag(001) respectively). The colour code indicates the positions in (**b**) where the spectra were acquired. Insets show the in-gap (${V}_{b}=5$ mV) constant-height tunnelling current image of the GNRs on MgO. The green curve is an average of spectra taken along the dashed line in (**b**). **g)** High resolution *dI/dV* spectrum within the region enclosed by the dotted rectangle in (**f**) (stabilization ${V}_{b}=$0.5 V, ${I}_{t}=$200 pA and ${V}_{{\mathrm{mod}}}=$0.5 mV r.m.s.).

Chiral graphene nanoribbons (*m,n,w*)-GNRs grow with their longitudinal axis along a vector (*m*, *n*) of the graphene lattice, enclosing *w* C-C pairs among them. We work with (3,1,8)- and (3,2,8)-GNRs, whose edge consists in an alternating sequence of three zig-zag graphene vectors and arm-chair segments (Fig. 1a). Due to this geomtery, they exhibit edge states and a symmetry-protected topological (SPT) energy gap, giving rise to zero-energy SPT end states at their termini^17^. In the presence of electronic correlations, both edge and SPT states can host spins with different degree of delocalization. However, on the rather electrophilic substrates like Au(111) and related intermetallic surface GdAu_2_, we found slight doping of holes and electrons respectively^17,19^, with negligible correlation gaps.

We synthesized (3,*n*,8)-GNRs on Ag(001) by thermally activated Ullmann coupling and subsequent cyclodehydrogenation of precursors **1** and **2**^17,19^, leading to GNRs with *n* = 1 and 2, respectively (see Fig. 1a, b and Supplementary Fig. 2) with varying lengths between *L* = 2 to 22 precursor units (PU). High-resolution constant height current images (Supplementary Fig. 2) with CO-functionalized tips confirm the correct structure of the GNRs. Differential conductance (*dI/dV*) spectra on the GNRs over the Ag(001) surface display a set of broad peaks around the Fermi level (Fig. 1f) that, in anticipation to our later results, can be assigned to QW states stemming from the confinement of the conduction band localized at the edges^17,19^. The typical full-width at half-maximum (FWHM) of these resonances varies from ∼50 meV to 100 meV (see Fig. 1f and Supplementary Fig. 4).

The GNRs were transferred to the previously grown MgO monolayer patches by means of lateral manipulation with the STM tip (see Fig. 1c–e and Methods). This technique involves smaller forces and is less invasive than vertical manipulation mode that is necessary to relocate GNRs onto other insulators like NaCl. Lateral manipulation is possible because the MgO ML islands are embedded on the Ag surface, and thus GNRs are synthesized right on the plane of the MgO surface (see Supplementary Fig. 3). The GNRs over the MgO island exhibit a distinct change in STM topographic images (Fig. 1b) and, more importantly, their electronic structure changes drastically. As shown in Fig. 1f, g for (3,*n*,8)-GNRs with *L* = 5 PU, the molecular resonances are much sharper, featuring linewidths of the order of 1 meV (Supplementary Fig. 5 illustrates how it is determined). Each resonance appears followed by two satellite peaks at 7.6 and 76 mV distance (independently of the GNR length) that correspond to the excitation of Franck-Condon (FC) resonances (see Supplementary Figs. 6 and 13), in analogy with previous studies of individual molecules and GNRs weakly coupled to metal surfaces^33–35^.

**Fig. 2:**
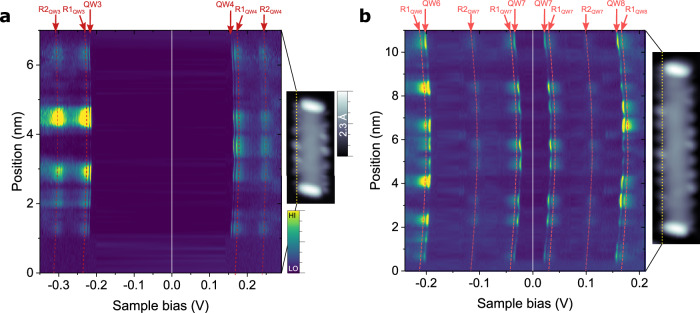
Detailed electronic structure of edge state in GNRs/MgO. Stack plots of *dI/dV* spectroscopy curves taken along the chiral edge of (3,1,8)-GNRs with *L* = 6 (**a**) and *L* = 11 (**b**). Insets show 3 nm wide topography images (*V*_b_ = 0.5 V, *I*_t_ = 50 pA). Dotted yellow lines indicate the positions where spectroscopy was retrieved. Spectroscopy parameters: stabilization *V*_b_ = 0.5 V and *I*_t_ = 200 pA, *V*_mod_ = 1 mV rms, *T* = 1.2 K and 4.3 K for (**a**) and (**b**) respectively. n^th^ order QW states are labelled as QWn. Reddish dashed lines are a guide to the eye of the position dependent energy of their associated Franck-Condon resonances (R*m*_QW*n*_: m^th^ replica of the n^th^ QW state, see Supplementary Fig. 6).

**Fig. 3:**
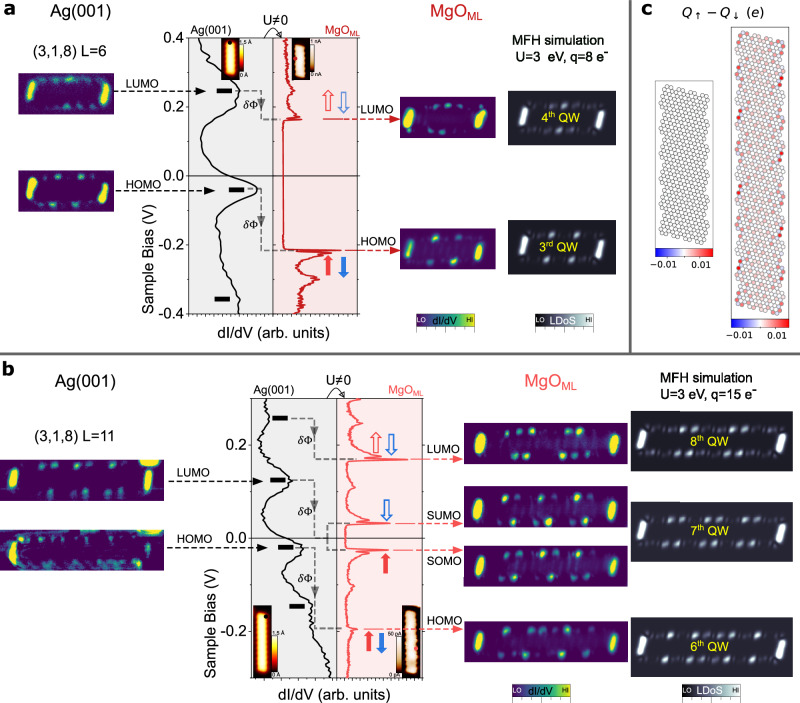
Even/odd occupancy of discrete QW states in (3,1,8)-GNRs. **a**, **b** *dI*/dV point spectra of L = 6 and 11 GNRs respectively taken at the positions marked in the insets. Images on the left column are experimental constant current *dI/dV* maps at the indicated energies on Ag(001). Images on the right column are constant height *dI/dV* maps on MgO_ML_ and theoretical LDOS simulations (see Methods) of the QW states indicated by the yellow labels. Black[red] dashed arrows mark the energy at which the different QW states appear on Ag(001)[MgO_ML_], connecting the spectroscopic features with their corresponding maps. For *L* = 11 (**b**), the 6^th^ QW state is close enough to the Fermi level so that the finite *U* plays a significant role, splitting the two spin channels. The red/blue filled[empty] arrows represent the spin of the single electron occupied[unoccupied] states. **c** Calculated MFH spin polarization projected on the C sites (see Methods) for *L* = 6 and *L* = 11 GNRs with *U* = 3.0 eV and charge states of 8 and 15 electrons, respectively. STM parameters: Insets in (**a**) and (**b**) display the topography at *V*_b_ = 0.5 V and *I*_t_ = 50 pA in the case of Ag(001) and constant height in-gap current at *V*_b_ = 2 mV in the case of MgO_ML_. *dI/dV* spectroscopy parameters in (**a**) and (**b**): stabilization at 0.5 V/100 pA (*V*_mod_ = 5-8 mV) for Ag(001) and 0.5 V/200 pA (*V*_mod_ = 1 mV) for MgO_ML_. Parameters for *dI/dV* mapping: on Ag(001) constant current maps are taken at *V*_b_/*I*_t_ = 210 mV/200 pA (QW4) and −72 mV/200 pA (QW3) with *V*_mod_ = 5 mV for *L* = 6 (**a**), and 123 mV/100 pA (QW7) and −30 mV/100 pA (QW6) with *V*_mod_ = 8 mV for *L* = 11 (**b**); constant height maps on MgO_ML_ are taken after opening the feedback at the GNRs’ centre with regulation set points of −200 mV/200 pA (QW4) and 150 mV/150 pA (QW3) with *V*_mod_ = 1 mV for *L* = 6 (**a**), and of 50 mV/20[100/5] pA (QW8[QW7/QW6]) with *V*_mod_ = 1 mV for *L* = 11 (b). *T* = 1.2 K. All experimental main images have 3 nm height, while the all theoretical images have 4.4 nm height. Insets are 3 nm wide.

To elucidate the orbital character of the sharp GNR resonances on MgO, we acquired *dI/dV* spectroscopy curves along the GNRs edges. Figure 2a and b show stacked spectroscopy plots as a function of bias and position along the edge for (3,1,8)-GNRs with *L* = 6 and 11, respectively. In *L* = 6 (Figs. 2a, 3a) we observe two QW states (each one flanked by their FC replica) separated by a large energy gap of 380 meV. Their spatial distribution shows conductance maxima distributed along the edge, with different number and position of nodal planes on either side of the Fermi level, as would be the case for a discretization gap of the conduction band. In the case of *L* = 11, there are four different QW states within the same energy window (Figs. 2b, 3b), what is consistent with the larger GNR length. Notably, for *L* = 11, the frontier QW states around the Fermi level exhibit identical intensity profiles along the edge. As will be discussed below, this feature is characteristic of a correlation gap, as opposed to the discretization gap proposed for *L* = 6.

We performed a quantitative assignment of QW order to the resonances on both Ag(001) and MgO_ML_ by comparing in Fig. 3a, b their experimental spatial distribution with MFH calculations of the local density of states (LDOS) (see Methods). As shown in Supplementary Fig. 10, due to the intrinsic peak broadening on the metal, the QW states close to the SPT state with small energy spacing cannot be individually resolved. Still, the position of the SPT state can be tracked down as a broad occupied peak, which lies well below the Fermi level on Ag(001). On the other hand, the QW states around the Fermi level can be readily identified. For *L* = 6 (3,1,8)-GNR on Ag(001), we experimentally find an occupied state at −42 mV and a fully unoccupied state at 245 mV (Fig. 3a, left). MFH results for the LDOS of the 3^rd^ and 4^th^ QW states reproduce the experimental *dI/dV* maps of both peaks (Fig. 3a, right). Taking into account that each QW state can accommodate two electrons (with opposite spin), plus two additional electrons in the SPT state (one at each ribbon end), we conclude that this ribbon hosts a fractional charge state of slightly less than *q* = 8 e^−^ excess electrons relative to the charge-neutral case. Note that the 3^rd^ QW state is still partially unoccupied due to Fermi level pinning. When the GNR is transferred onto the MgO island, all states experience an energy downshift, whereas their intensity distribution still corresponds to the LDOS of the same QW states (Fig. 3a). The 3^rd^ QW state shifts down to −216 mV, and the 4^th^ QW state appears at 163 mV. Therefore, the GNR retains approximately the same occupancy, although in this case, the charge doping is exactly *q* = 8 e^−^, because there is no in-gap spectral weight. As sketched in Fig. 3a, the n-doping on the oxide layer is a consequence of the reduced work function of MgO/Ag(001) relative to the bare Ag(001)^36^, which we have determined as $\delta \Phi$_[Ag-MgO]_=0.63 eV from the analysis of their respective field emission resonances (see Supplementary Fig. 7).

The very narrow line-shape of the molecular resonances on MgO, and the appearance of FC resonances that require transitions to long-lived excited molecular states^33^, points to a very efficient electronic decoupling from the metal substrate. In this context, one would expect enhanced e-e interactions^24,37^. This is the case of the (3,1,8)-GNR with *L* = 11 shown in Fig. 3b, which displays a qualitatively different behaviour than the *L* = 6 case. On Ag(001) we find a partially occupied state at −23 mV and a fully unoccupied state at 123 mV. MFH simulations (see also Supplementary Fig. 8) reveal that they correspond to the 6^th^ and 7^th^ QW states. Taking into account the two additional electrons hosted by the SPT state, we conclude a charge state of slightly less than *q* = 14 e^−^. On MgO, all states experience again an energy downshift. The 6^th^ QW state shifts in energy down to −200 mV. The 8^th^ QW state, which was barely resolved on Ag(001) at around 250 mV, is now prominent at 171 mV. Remarkably, the 7^th^ QW appears split in two states with identical spatial distribution at −30 mV and 27 mV. Since only one of them is occupied, we conclude an odd occupancy of exactly *q* = 15 e^−^, which implies one singly occupied and one singly unoccupied frontier state with opposite spin, as depicted by the arrows in Fig. 3b. This remarkable large negative charge is in line with the huge energy shift of the SPT state of −650 meV, which would mark the charge neutrality point in the absence of charge transfer from the substrate (see Supplementary Fig. 11 and the discussion about high bias range spectroscopy in Supplementary Section III).

The work function reduction in the MgO (δΦ_[Ag-MgO]_, see Supplementary Fig. 7) acts here as a gating potential for the QW states (Supplementary Table 1 and Fig. 9)^36^. We represent its associated energy downshift in Fig. 3a, b by dashed grey vertical arrows. Many-body correlations are taken into account in the MFH model by the on-site Coulomb repulsion, *U*. Its effect for the 7^th^ QW state of *L* = 11 at half-filling as *U* increases is to induce a splitting of the two spin channels (experimental value in Fig. 3b of ∼50 meV). Due to the confinement of the edge state band^17,19^, if the 7^th^ QW state is sufficiently close to the Fermi level for *U* = 0, small *U* values will cause the population of one additional state with one electron from the Ag(001) reservoir. Within the MFH model, this leads to a total spin-½ distributed as depicted by the simulation of the spin density shown in Fig. 3c. In contrast, for *L* = 6, electron correlations (*U* > 0) renormalize the eigenenergies (∼10 meV for *U* = 3 eV), but the highest occupied molecular state remains deep below the Fermi level, making the double occupancy (i.e. closed shell configuration) energetically favourable and rendering a non-magnetic ground state.

**Fig. 4:**
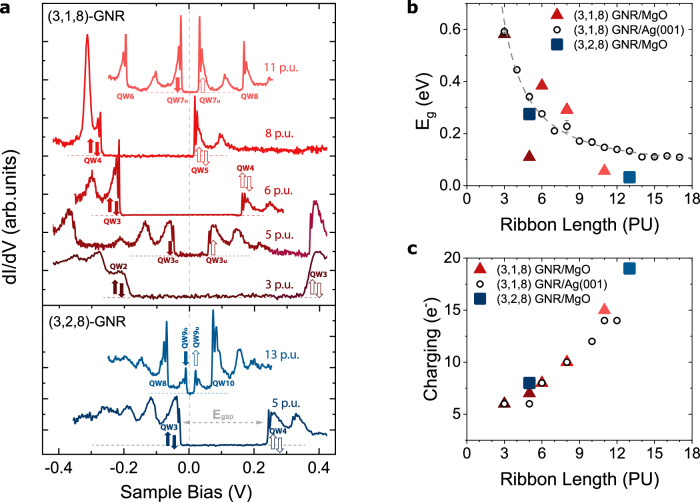
Energy gap across the Fermi level in (3,*n*,8)-GNRs repositioned on MgO_ML_. **a** Waterfall plot of *dI/dV* spectra (stabilization at ${V}_{b}=$0.5 V, ${I}_{t}=$200 pA; ${V}_{{\mathrm{mod}}}=1\,$ mV r.m.s.; *T* = 1.2 K for *n* = 1 and 4.3 K for *n* = 2) of several (3,*n*,8)-GNRs showing the non-monotonous behaviour of the gap across the Fermi level. The acquisition position in the case of *L* = 6,11 is shown in the insets of Fig. 3a, b. Filled/empty arrows represent occupied/unoccupied single electron states with well defined ${S}_{z}$ quantum number. For *L* = 8, the broad peak at −0.3 V is ascribed to the charging of a point defect in proximity (Supplementary Figure 14). **b** Evolution of the gap as a function of the GNR length of (3,1,8)-GNRs on Ag(001) –empty circles, error bars are derived from measurements in 2 or 3 GNRs of the same length– and several representative examples of (3,1,8)/(3,2,8)-GNRs on MgO_ML_ –triangles/squares–. The dashed line is an asymptotic fit proportional to ${L}^{-3/2}.$ **c** Exact charge state of (3,1,8)/(3,2,8)-GNRs on MgO monolayer and approximate charge state of (3,1,8)-GNRs on Ag(001), see Supplementary Table 1 for further details.

All (3,1,8)- and (3,2,8)-GNRs investigated here can be classified into the two categories shown in Fig. 3, i.e., either odd occupation with identical QW states around the Fermi level, or even occupation with different QW states (see Supplementary Fig. 8). Figure 4a, b illustrate an interesting anomaly in the gap across the Fermi level (*E*_*g*_) of (3,*n*,8)-GNRs on MgO: its size does not vary monotonically as a function of length. For instance, the gap for *n* = 1 and *L* = 5 is about 100 meV, whereas for just one more PU (*L* = 6) the gap increases abruptly to ∼400 meV, to experience again a substantial drop between *L* = 8 (*E*_*g*_~ 300 meV) and *L* = 11 (*E*_*g*_~ 50 meV). This is in contrast with the evolution of the GNRs’ gap on Ag(001), which follows the expected asymptotic decay with increasing length of a particle-in-a-box (Fig. 4b and Supplementary Fig. 4). On MgO, some (3,*n*,8)-GNRs exhibit similar gaps to the ones on Ag(001), while others have a much reduced value. Figure 4c shows the excess charge (*q*) in all GNRs determined by means of the same procedure as for *L* = 6 and *L* = 11 GNRs above. The anomalously small values of *E*_*g*_ (Fig. 4b, c) are univocally linked to frontier states with identical spatial distribution (see Supplementary Fig. 8) and, hence, to odd occupations. These GNRs have, thereby, open-shell character of the edge state and non-zero total spin.

**Fig. 5:**
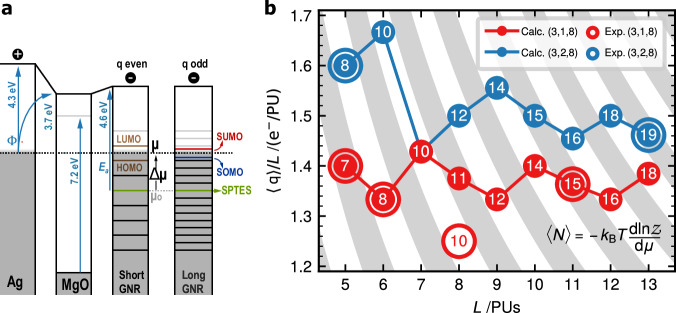
Comparison of experimental electron occupation and theoretical calculations. **a** Energy level alignment scheme for short even- and long odd-integer charged GNRs on top of a MgO layer (band gap 7.2 eV^50^), respectively. The neutral level of the GNRs is referenced by the binding energy of the two symmetry-protected topological end states (SPTEs). **b** Fit of the grand canonical charging model to the experimentally observed charge per precursor unit as a function of ribbon length for the two types of chiral GNRs. Stripes in grey correspond to odd-integer excess charge. The number in each data point is the obtained $\left\langle q\right\rangle$ value.

To address this scenario, we developed a theoretical model accounting for electron doping as a function of length and chirality. When molecular species lie on substrates with work function Φ lower than their electron affinity *E*_*a*_, an interfacial charge redistribution takes place, resulting in the electron accumulation into unoccupied molecular states and the build-up a local interface dipole with associated potential energy *U*_*d*_ across the dielectric spacer, which opposes to charge transfer^38–40^. In equilibrium, the charge state of the molecule is determined by the shift of *μ* (the chemical potential at the metal’s Fermi level) from the value at which the molecule retains charge neutrality, *μ*_0_ (Fig. 5a).

1 $$\Delta {{\mu }}={{\mu }}-{{{\mu }}}_{0}={E}_{a}-\Phi -{U}_{d}$$

Since the Ag(001) work function (Φ_Ag(001)_ = 4.3 eV^37,39,41^) is smaller than the GNR’s electron affinity (comparable to the bulk work function of graphene, Φ_GNR_ ~ 4.61 eV^42^), the nanoribbons exhibit significant n-doping already on Ag(001). As shown in Supplementary Table 1, we find an average doping of 1.25 e^−^/PU for (3,1,8)-GNR on Ag(100), in close agreement with the previous reported value of 1.3 e^−^/PU on a similar system^43^. The addition of a MgO insulating layer further reduces the substrate’s work function via the pillow effect^37,44,45^ by δΦ = 0.63±0.12 eV (see Supplementary Fig. 7), to yield a rather low work function Φ_Ag/MgO_ ~ 3.7±0.1 eV. The MgO also suppresses the wave-function overlap between molecule and metal such that only integer charges are allowed. Depending on the alignment of *μ* with respect to the discrete molecular levels (Fig. 5a), the resulting charge state may be either even or odd.

To extract the magnitude and parity of the acquired excess charge (*q*), we represent each GNR in contact with the substrate bath by a chemical potential *μ* and temperature *T* in the grand canonical ensemble. We compute the internal energy of the various charge states of (3,*n*,8)-GNRs (*n* = 1,2) with increasing length *L* using the MFH model (see Supplementary Note 4) and obtain the mean number of excess electrons $\left\langle q\right\rangle$ for any given chemical potential *μ* (see Supplementary Fig. 12) via the relation

2 $$\left\langle q(\mu,L,T)\right\rangle=-{k}_{B}T\frac{d{{\mathcal{Z}}}(\mu,L,T)}{d\mu }$$

where *Z(μ,L,T)* is the grand canonical partition function obtained by summing over all relevant charge states for each GNR (See Supplementary Note 4).

Our model reproduces that for a fixed chemical potential the excess charge increases with length. In fact, the addition of one additional PU increases *q* by either zero, one or two electrons, very much in line with the experimental observation of a non-integer mean value of *q/L*. This is due to the intrinsic evolution of the level spacing of the QW edges states of (3,*n*,8)-GNR with length and chirality. In the thermodynamic limit *L*→∞, $\left\langle q\right\rangle /L$ asymptotically approaches the intensive value corresponding the n-type carrier density of a one dimensional GNR gated by an electric potential $\Delta \mu /{|e|}$.

To obtain the local gating *Δμ* of each family of chiral ribbons on the MgO layer, we fit the experimental electron doping deduced in Figs. 3, 4 with predicted charges using *Δμ* as a length-independent free parameter. The results of the fit, shown in Fig. 5b, reproduce the charging pattern of (3,1,8)- and (3,2,8)-GNRs with *Δμ* = 0.51 eV and 0.49 eV, respectively (see Supplementary Fig. 12). The only experimental deviation is the case of (3,1,8)-GNR with *L* = 8, for which the model predicts the single occupancy of the 5^th^ QW state. Instead, this GNR appears on MgO charged with ten electrons, with its LUMO (the 5^th^ QW state) at only 17 meV above *E*_*F*_ (Fig. 4a and Supplementary Fig. 8), i.e., at the verge of single occupancy. This is likely due to the electrostatic potential emanating from nearby charged defects in the MgO lattice (see examples in Supplementary Figs. 14 and 15).

The determined *Δμ* value is about half of the ∼0.9 eV work function difference between MgO/Ag(001) and graphene. This can be attributed to the existence of a sizable interface dipole across the MgO layer, which conversely acts to lower the GNR’s charge state^40^. From Eq. (1), the electrostatic energy stored in the interface becomes ${U}_{d}$∼0.4 eV, which matches well with a plate capacitor model^40^ with excess charge lying mainly on the zig-zag edges of the GNRs.

In conclusion, when chiral GNRs are positioned on the MgO monolayer on Ag(001), the combination of its low work function and the electronic decoupling gives rise to quantized charge transfer to their edge states, whose occupation can be controlled by a modification of the length of just one PU. As a consequence, the Fermi level raises up to QW states that lie 300 to 650 meV above in the corresponding charge neutral GNR. Furthermore, the e-e correlations of extended GNR edge states on MgO are sufficiently large as to stabilize singly occupied QW edge states, leading to spin-1/2 quantum dot behaviour at the MFH level of theory, with spin-split frontier states. This is reminiscent of the single electron transistor behaviour of individually contacted GNRs^46^, but here the dot occupancy is controlled by the GNR length in addition to a gate voltage. It opens up possibilities for disruptive functional devices such as graphene-based spin qubits and spin-polarized field effect transistors.

Our experimental set up combines ample synthetic capabilities with the utmost sensitivity to the electronic structure. This opens up opportunities to design functional nanographenes with well-defined quantum states, unlike existing strategies to fabricate devices based on GNRs: the transfer of semiconducting GNR arrays to Si wafers, alumina^46^ and graphene^47^; the direct synthesis on oxide^25^ surfaces, the intercalation of non-metallic layers or functional groups^35^; and the manipulation onto thin layers of NaCl^12,24,26^.

For example, on MgO/Ag(001) the energy landscape determined by the electrostatic potential at the tip-sample gap can induce rigid shifts of the whole GNR spectrum of the order of tens of meV (Fig. 2a, b and Supplementary Note 6), or even switching between odd and even occupancy (Supplementary Fig. 15). This can be combined with atomic manipulation of the GNRs, leading to interacting quantum dots with controllable spin and charge states.

## Methods

### Sample preparation

Samples are prepared at a base pressure of 1 × 10^-10 ^mbar. The Ag(001) single crystal from SPL B.V. was cleaned by repeated Argon sputtering and annealing at 430 °C. MgO monolayer patches are grown by depositing Mg from a crucible (MBE Komponenten GmBh effusion cell) heated at 320 °C onto the clean Ag(001) held at a constant temperature of 390–400 °C in an O2 partial pressure of 1×10^-6 ^mbar. The growth rate of MgO under these conditions fluctuates between 0.5 to 0.1 ML/min. After deposition, we wait a time lapse of 30 min to properly pump down the residual O_2_ molecules in the chamber (*p* < 1×10^-9 ^mbar), and then we anneal the sample during 20 min at 390 °C with the purpose of healing the disorder at the edges of the MgO and decrease the number of point defects within the islands (see Supplemental Fig. 1). To synthesize the (3,*n*,1)-GNRs we sublimate onto the Ag(001) surface (already with the MgO patches on it) the precursor reactants **1** and **2** (Fig. 1a) that yield GNRs with *n* = 1 and *n* = 2 respectively and then perform a single annealing step at 345 °C during 15 min to achieve full cyclodehydrogenation of the GNRs (further details in Supplementary Experimental Methods). The synthesis and quality of the GNRs on Ag(001) with and without coexisting MgO monolayer islands is the same, with the only exception that in the presence of MgO, the GNRs are in average shorter, probably owing to the lower mobility of short precursor oligomers on the surfaces with lower available metallic area.

### Lateral atomic manipulation of GNRs

GNRs are relocated on the surface by approaching the tip to their arm-chair termini (where large electron density is typically concentrated near Fermi level) until attracting forces between the tip foremost atoms and the ribbon are large enough as to drag the ribbon jumping repeatedly below the tip as it moves laterally. This procedure is illustrated in Supplementary Fig. 3 and 4. To move the GNR across the Ag surface, the tip moves under closed feedback conditions at a tunnelling resistance given by the set point of 3 mV and 10–40 nA. To transfer the a GNR to a MgO patch in close proximity, we use instead open feedback conditions at a similar tunnelling set point of 1–3 mV and 10–60 nA.

### Scanning tunnelling microscopy and spectroscopy

All measurements have been performed at the SPECS-JT-STM of the Laboratory for Advanced Microscopy (University of Zaragoza). The whole system operates under ultra-high-vacuum conditions (1 × 10^-10 ^mbar). The tip is grounded and the tunnelling bias *V*_*b*_ is applied to the sample. Data has been taken at *T* = 1.2 K unless stated otherwise. Differential tunnelling conductance *dI/dV* is acquired using a lock-in amplifier at a frequency of 973 Hz and r.m.s. modulation given by *V*_mod_. STM images and *dI/dV* maps were taken either in constant height or in constant current mode, using a stabilization distance determined by the set point indicated at the corresponding caption for each data set. Tips are prepared by electrochemical etching of W wires and subsequent field emission cleaning (120 V, 1 μA, 30 min) at the STM head. CO functionalization of the tip is achieved by controlled approach of the tip to a CO adsorbed on the surface at |*V*_*b*_ | <5 mV until a sudden jump in the current is detected.

### Mean-field Hubbard calculations

The electronic structure and magnetic ground state of GNRs is well captured by the Hubbard model^48^. By employing a mean-field approach^49^, we solve for the ground state and obtain eigen-functions and -energies of the π-electrons, using the same Hamiltonian parametrization as in Refs. ^17,19^, which has been previously found to reproduce the experimental band gaps in GNRs. We choose a Hubbard parameter of *U* = 3 eV to capture the observed SOMO-SUMO splitting as a function of length and chirality. Differential conductance maps are simulated by squaring a linear combination of p_z_ orbitals on the lattice at a height of 1.8 nm.

## Supplementary information

Supplementary Information

Transparent Peer Review file

## Source data

Source Data

## Data Availability

The data generated in this study have been deposited at the public repository DIGITAL.CSIC with permanent URL 10.20350/digitalCSIC/17248. Source data are provided with this paper.

## Code availability

Computer code used to solve the Hubbard Hamiltonian and simulate the images of the density of states is available at https://zenodo.org/records/4748765. The extension of this code to calculate the charge excess in the grand canonical ensemble is available at 10.5281/zenodo.15165880.
