# Supplementary material for: Systematic modulation of charge and spin in graphene nanoribbons on MgO
Source: Nat Commun. 2025 Jul 1;16:5632. doi: 10.1038/s41467-025-60767-5 (PMC12218530; doi:10.1038/s41467-025-60767-5)
Supplement: Supplementary file 1 — Supplementary Information [file 41467_2025_60767_MOESM1_ESM.pdf]

Supplementary Information for:

Systematic Modulation of Charge and Spin in Graphene Nanoribbons on MgO

Amelia Domínguez-Celorrio[1,2,3,†], Leonard Edens[4,†], Sofía Sanz[5], Manuel Vilas-Varela[6], Jose Martinez-Castro[7], Diego Peña[6], Véronique Langlais[8], Thomas Frederiksen[5,9], José I. Pascual[4,9], and David Serrate[1,10,11*]

[1]Insituto de Nanociencia y Materiales de Aragón (INMA), CSIC-Universidad de Zaragoza, Zaragoza, E-50009, Spain
[2]School of Physics and Astronomy, Monash University, Clayton, VIC 3800, Australia
[3]ARC Centre for Future Low Energy Electronics Technologies, Monash University, Clayton, VIC 3800, Australia
[4]CIC NanoGUNE BRTA, San Sebastián, E-20018, Spain
[5]Donostia International Physics Center, San Sebastián, E-20018, Spain.
[6]Centro Singular de Investigación en Química Bilóxica e Materiais Moleculares (CiQUS) and Departamento de Química Orgánica, Universidade de Santiago de Compostela, Santiago de Compostela, E-15782, Spain.
[7]Peter Grünberg Institut (PGI-3), Forschungszentrum Jülich, 52425 Jülich, Germany
[8]Centre d'Elaboration de Materiaux et d'Etudes Structurales, CNRS, Toulouse, F-31055 France
[9]Ikerbasque, Basque Foundation for Science, Bilbao, E-48013, Spain.
[10]Departamento de Física de la Materia Condensada, Universidad de Zaragoza, Zaragoza, E-50009, Spain
[11]Laboratorio de Microscopias Avanzadas (LMA), Universidad de Zaragoza, Zaragoza, E-50018, Spain.

[†]These authors contributed equally
*email: serrate@unizar.es

Table of Contents:

I. Supplementary Methods.

II. Supplementary Note 2.

III. Supplementary Note 3.

IV. Supplementary Note 4.

V. Supplementary Note 5.

VI. Supplementary Note 6.

VII. Supplementary References.

I. Supplementary Methods

MgO ultrathin patches on Ag(001) are grown by depositing Mg from an alumina crucible) heated at 320-330 ºC onto the clean Ag(001) held at a constant temperature of 390-400 ºC in an O2 partial pressure of 1×10^{-6} mbar. The growth rate of MgO under these conditions fluctuates between 0.5 to 0.1 ML/min. After deposition, we wait a time lapse of 30 min to properly pump down the residual O_2 molecules in the chamber ($p<1\times10^{-9}$ mbar), and then we anneal the sample during 15-20 min at 390 ºC with the purpose of healing the disorder at the edges of the MgO and decrease the number of point defects within the islands (see Supplementary Fig. 1).

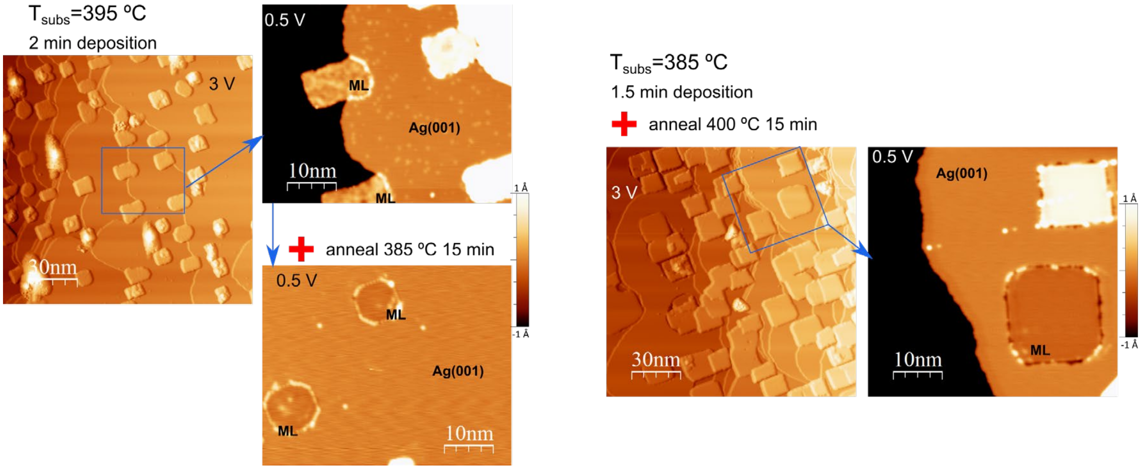

Supplementary Figure 1.- Growth parameters of MgO ultrathin islands on Ag(001). STM images (150x150 nm) obtained at a sample bias of 3 V are overviews of two samples grown under similar conditions (fake colour scale to better appreciate the island roughness). Left panel correspond to the as-grown sample while the right panel corresponds to a sample which was post-annealed a 400ºC. The zoomed regions are imaged at 0.5 V. This bias allows us to distinguish between embedded monolayer islands (labelled as ML) and other thicknesses. The ML islands become nearly defect free after post-annealing under UHV conditions.

(3,*n*,1)-GNRs synthesis starts by sublimating precursors **1** and **2** (Supp. Fig. 2a and 2e) onto the Ag(001) surface where MgO monolayer (MgO_{ML}) patches have been previously grown, for *n*=1 and *n*=2 respectively. Ullmann coupling of precursor **1** takes place at room temperature as they reach the Ag(001) surface. As shown in Supplementary Fig. 2b, the precursor units assemble as short oligomers, mostly attached to the edges of Ag terraces. Upon annealing to 300 ºC (Supplementary Fig. 2c), we distinguish a clear change in the morphology of the sample with longer and ordered chains in islands. The distance between the protrusions shown in Supp. Fig. 2c is 8.7(1) Å, which corresponds with the expected periodicity of poly-(**1**) chains. Mild annealing processes of poly-(**1**) samples above 300 ºC led to partial cyclodehydrogenation (CDH) of the polymeric chains (Supplementary Fig. 2c). Fully planarized (3,1,8)-GNRs form after 15 minutes at 345 ºC (Supplementary Fig. 2d).

Similarly, as illustrated by Supplementary Fig. 2e, precursor **2** also undergoes Ullmann coupling on Ag(001) at room temperature. In this case, densely packed and ordered poly-(**2**) chains appear without any additional annealing for sufficiently large coverages (as in the left side terrace of Supplementary Fig. 2f). The characteristic period of these chains is 10.9(1) Å, which corresponds with the expected periodicity of poly-(**2**) chains (Supplementary Fig. 2g). Annealing of poly-(**2**) samples at 345 ºC during 15 minutes led, as in the case of precursor **1**, to a complete CDH and the formation of (3,2,8)-GNRs (Supplementary Fig. 2h).

The synthesis and quality of the GNRs on Ag(001) with and without coexisting MgO_{ML} islands is the same, with the only exception that in the presence of MgO, the GNRs are in average shorter, probably owing to the lower mobility of the shorter precursor oligomers on the surfaces with lower available metallic area.

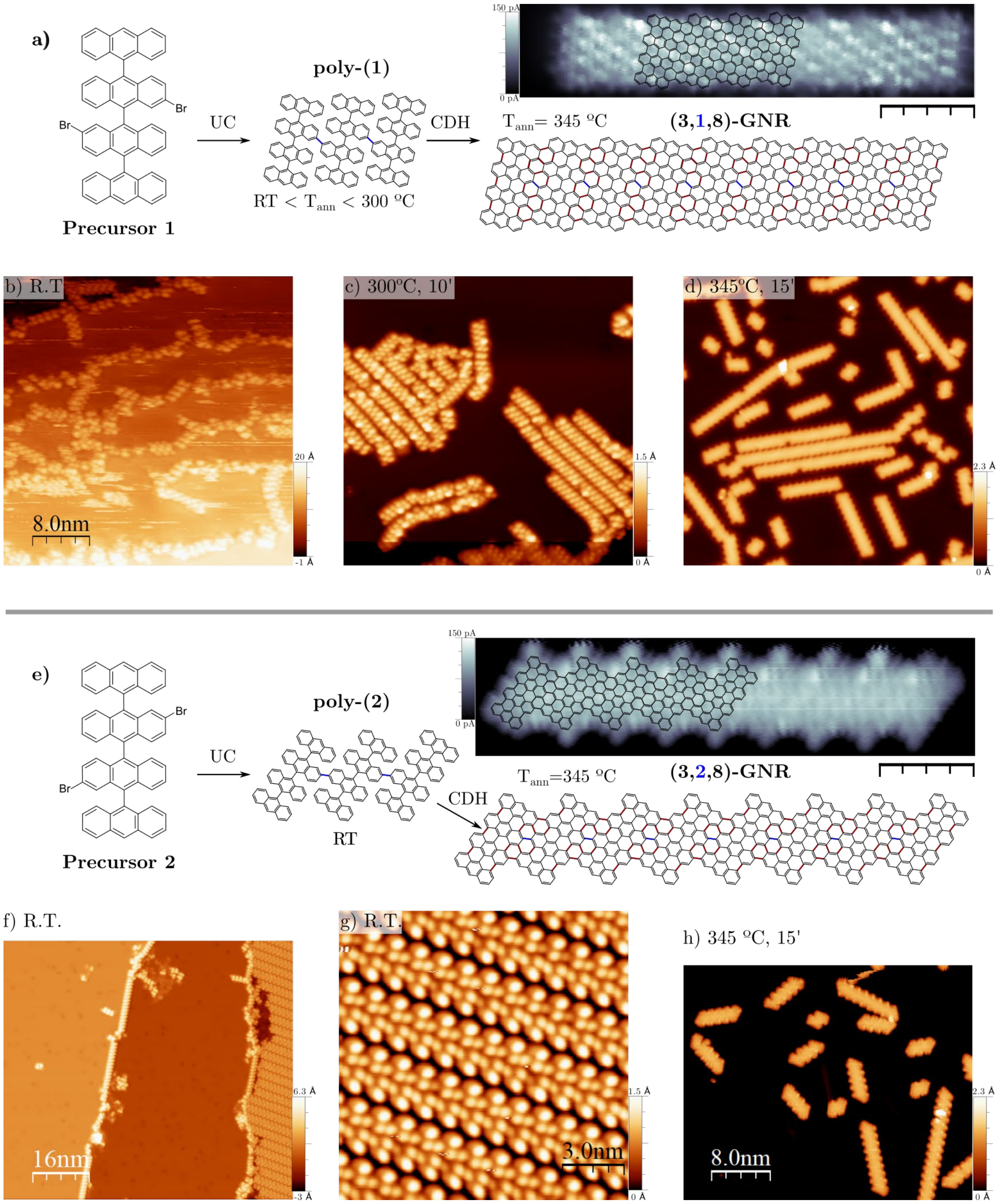

Supplementary Figure 2.- On-surface synthesis details of (3,*n*,8)-GNRs on Ag(001). Panels (a-d) correspond to n=1 and panels (e-h) to n=2. Panels (a) and (e) show the reaction schemes of precursors **1** and **2** respectively showing that Ullmann polymerization takes place already at room temperature, and that short annealing of the polymeric chains at 345 ºC gives rise to a complete cyclodehydrogenation (CDH) in both cases. Regulation V_b of STM topographies is 0.5 V in panels (b-d) , 1.8 V in panel (f), 0.05 V in panel (g), and 0.5 V in panel (h). High resolution insets in (a) and (e) are constant height tunnelling current images with functionalized tips taken at 1.5 mV and 500 mV for (3,1,8)- and (3,2,8)-GNRs respectively. Black scale bar in (a) and (e) is 2 nm. Scale bar is the same for panels (b), (c) and (d).

The direct growth on MgO surfaces is completely discarded, because the aromatic precursors do not adsorb on MgO at room temperature or higher (heating is absolutely necessary to induce dehalogenation). Furthermore, we are not aware of dry or wet transfer stamp techniques from catalytic surfaces to MgO films.

We have also explored the MgO intercalation after the growth of (3,1,8)-GNRs on clean Ag(001). We deposited directly MgO at $p_{O2}=1\times10^{-6}$ mbar with the GNR/Ag(001) held at 220 ºC, a temperature low enough as to preserve the GNRs integrity, and sufficiently high as to form MgO_{ML}. The edes of the GNR become decorated with multiple defects. The strategy of depositing Mg and post oxidizing it in a second step does not work either. Metallic Mg intercalates below the GNRs forming a kind of alloyed surface. However, subsequent exposure to O_2 results again in defective GNR edges, as found previously for 5-AGNRs [1].

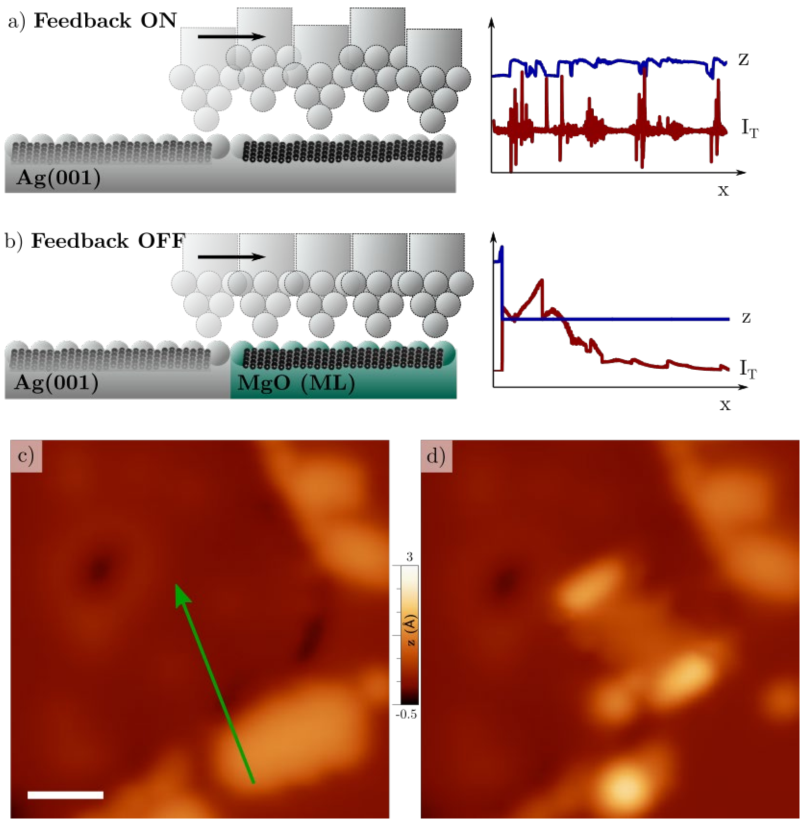

Supplementary Figure 3.- Lateral atomic manipulation of GNRs in pulling mode. (a) Sketch and manipulation traces of GNR on Ag(001). The tip exerts an attracting force over the termini of a GNR and moves laterally in constant current mode (feedback on). I_T is the tunnelling current and z the vertical position of the tip. (b) Sketch and manipulation traces of the transfer of a GNR from across the Ag-MgO boundary. The tip exerts an attracting force over the termini of a GNR and moves in constant height mode (feedback off) to avoid the abrupt jump down that would occur if the feedback was active. (c-d) Example of the successful transfer of a L=3 (3,1,8)-GNR. The traces in (a) and (b) correspond to exactly this example, which happened to be the first successful transfer in our project. White scale bar is 1.5 nm.

Therefore, we decided to resort to the relocation of the GNR by lateral atomic manipulation. Supplementary Fig. 3 shows schematically the manipulation procedure, together with manipulation traces characteristic of relocations in the Ag surface, and the transfer of the GNR to the MgO island across the MgO-Ag boundary. Lateral manipulation is performed in pulling regime with a gap resistance of the order of 10-50 nA at 3-5 mV bias

(sketch of the process in Supplementary Fig. 3a). The preferred contact points to trap the ribbon with the tip force field are the arm-chair termini, which is in line with the large density of states observed at this position (all QW states of the discretized conduction band display strog intensity at the termini, see for instance Supplementary Fig. 8). In average, the manipulation throughout the Ag surface is relatively simple and comparable with the case of individual 3d atoms on coinage metals. On the contrary, the success rate of the transfer from Ag to a MgO patch is much lower (sketch of the process in Supplementary Fig. 3b), and has to be performed under constant height conditions.

II. Supplementary Note 2.

Supplementary Fig. 4 shows, for (3,1,8)-GNRs on Ag(001), the gradual closing of the gap between the first fully unoccupied quantum well (QW) state and the immediately preceding in energy QW state. As the energy spacing between adjacent QW states decreases for increasing length *L*, the associated *dI/dV* resonances become slightly narrower. STM images on the left side of Supp. Fig. 4 illustrate the distinct change in apparent shape of the GNRs as they are stepwise introduced onto the MgO_{ML} patch.

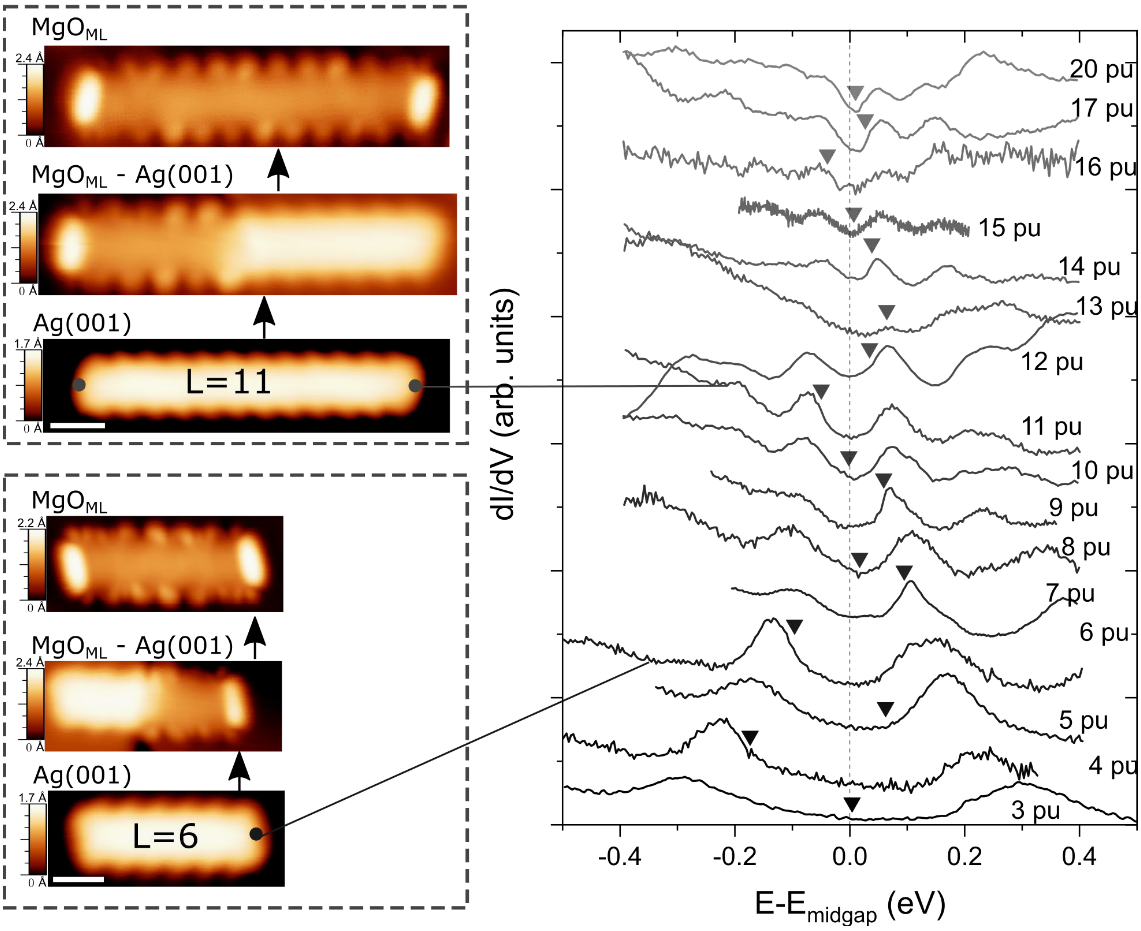

Supplementary Figure 4.- *dI/dV* of (3,1,8)-GNRs on Ag001 for several lengths. Topography and spectroscopy stabilization set points are 0.5 V and 50 pA. Lock-in modulation 5 mV r.m.s. Spectra have been vertically offseted and normalized for the sake of clarity. In the abscissa axis we represent the energy difference with respect to the midgap energy, E_{midgap}. rather than the traditional convention of referring energy with respect to Fermi level. E_{midgap} is defined as the middle point between the central energy of the first unoccupied (or partially unoccupied) state and that of the immediately preceding state in energy. The experimental Fermi level in each spectrum is indicated by triangles. In this way, the evolution of the gap between QW states nearest to Fermi level can be better appreciated. The left column shows topography images of (3,1,8)-GNRs on Ag(001) with L=6 and 11 and, subsequently in the vertical direction, the same GNRs after lateral manipulation: partially and fully inserted in the MgO_{ML}. The black circles mark the position where the corresponding *dI/dV* spectra were acquired. The spectra of the other ribbons were also taken at the arm-chair termini, where all QW states feature some intensity. Nevertheless, QW states at the same set of energies can be detected along the chiral edge, though they are often much less intense and not all of them manifest in the same position as a consequence of their intrinsic intensity pattern. White scale bars: 1.5 nm

One of the most striking changes observed on the MgO is the extremely low linewidth of the QW resonances. We have studied their full width at half maximum (FWHM) and the results are presented in Supplementary Fig. 5. At the lowest experimental temperature T=1.13 K the linewidth of the 3rd QW state of L=5 (3,1,8)-GNR decreases down to 1.3 mV as the lock-in modulation amplitude is reduced stepwise to values smaller than $k_B T/|e|$~0.1 mV.

2

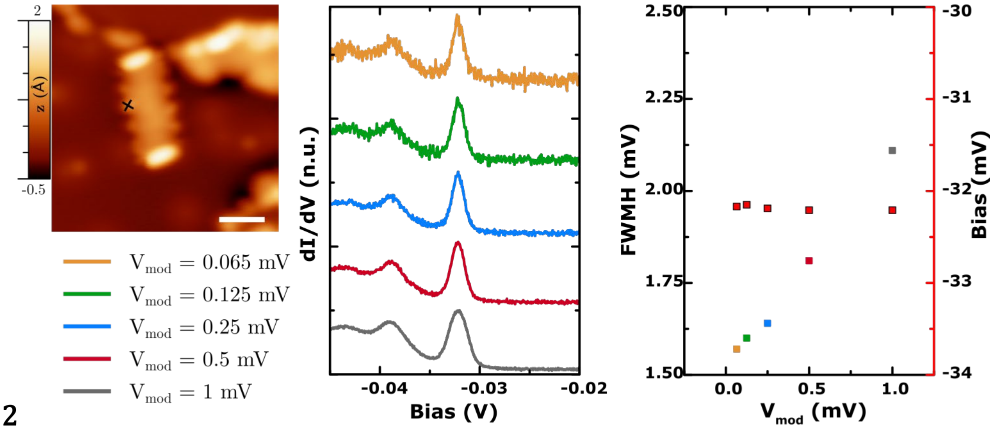

Supplementary Figure 5.- Determination of the linewidth of molecular resonances on MgO. STM topography image of a L = 5 (3,1,8)-GNRs on MgO_{ML} (0.5 V, 200 pA, T = 1.1 K, scale bar = 2 nm) and *dI/dV* point spectra recorded at the position of the cross with varying V_{mod} between 1 and 0.065 mV r.m.s. The left panel shows the experimentally determined FWHM (left axis, colour coded squares) and bias peak values (right axis, red squares) as a function of V_{mod}.

These sharp resonances are always accompanied by broader peaks that appear at 7.5 mV and 76 mV (see Supplementary Fig. 6a-c). This separation is constant throughout the GNR and independent of its length. Constant height *dI/dV* maps of the principal resonance (P, the sharp one) and their replicas (R) unveil that they have identical spatial distribution (Supp. Fig. 6a). This overall behaviour is characteristic of vibrational Franc Condon resonances excited as a consequence of long lived ionic GNR states as electrons or holes are injected into the QW states during the tunnelling process [2–4].

We have performed atomistic simulations the vibrational spectra of GNRs on MgO monolayer using harmonic forcefields (see computational details and analysis in Supplementary Theoretical Methods section IV.2). Our analysis suggests that the satellite peaks experimentally observed in dI/dV and labelled as R_1 and R_2 are, respectively, ascribed to the excitation of the external out-of-plane vibration of whole GNR, and to the out-of-plane H motion. In spite the external origin of the FC resonances, the associated modes happen to be nearly independent of the GNR length, in good agreement with the calculations.

As shown in Supplementary Fig. 6b-c, the vibrational excitations thresholds of $R_1$and R_2 are the same for occupied and unoccupied states, and are also the same for QW resonances of different order. They also keep the same values irrespectively of the open or closed shell state of the GNRs (cf. for instance with the example of the L=5 GNR in Supp. Fig. 6). This indicates that these vibrations are an intrinsic property of the GNR geometry and adhesion to the MgO, do not depend on the charge or spin states, and can be excited by tunnelling electrons to/from any molecular resonance.

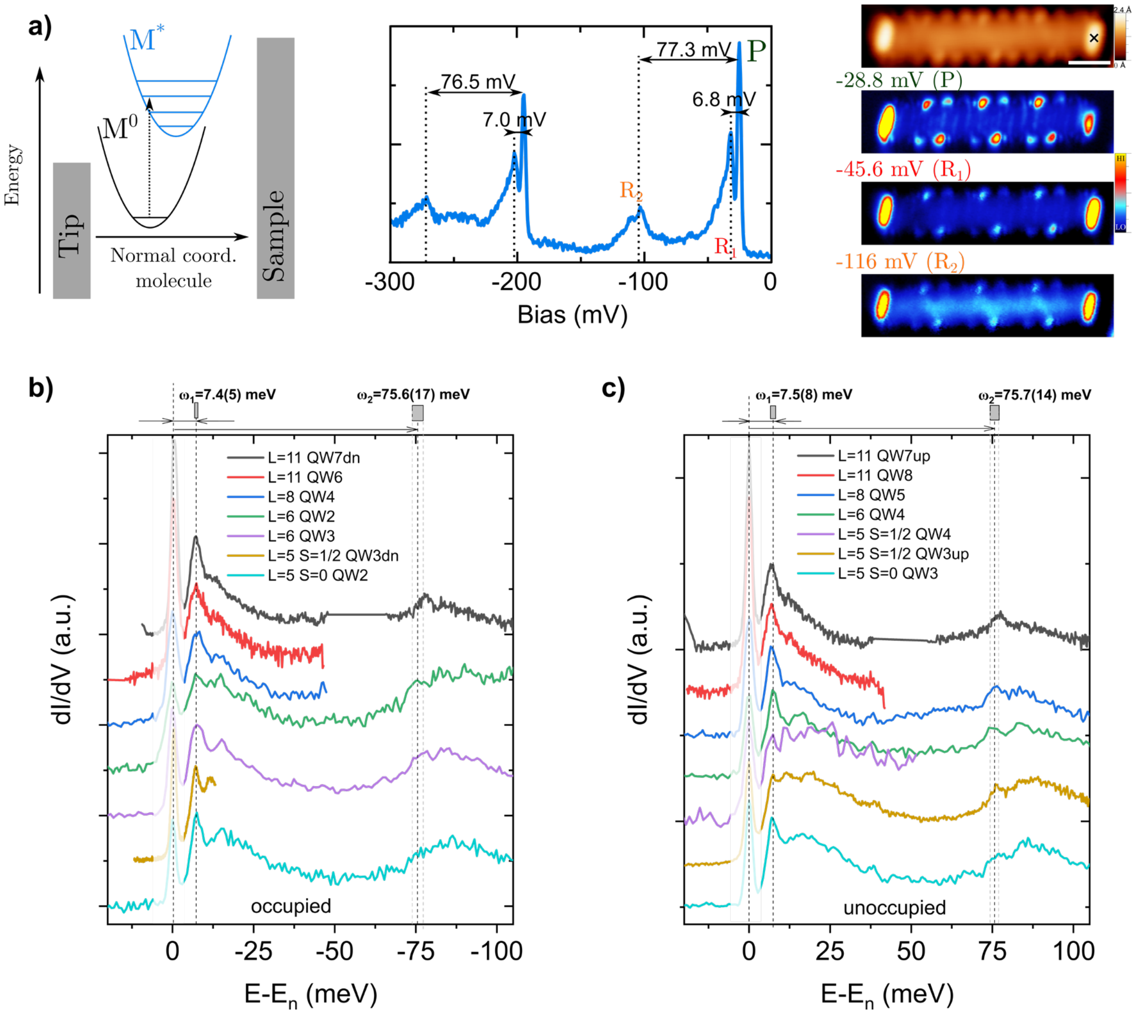

Supplementary Figure 6. Frank-Condon (FC) resonances and experimental vibrational density of states. (a) Sketch of Franck-Condon (FC) mechanism in an STM junction (adapted from ref. [2]) and example of *dI/dV* spectra with satellite resonances recorded for the (3,1,8)-GNR with *L*=11. The FC resonances are labelled as P, for the main peak, and R_1 and R_2 for the satellite peaks. V_{mod}=1 mV r.m.s. The right panel displays the topography of the GNR (the cross indicates the position for spectroscopy) and the constant height *dI/dV* maps recorded at the energies of the main and satellite peaks observed. White scale bar: 2nm. Notice that all three peaks exhibit identical spatial distribution. Set point for topography, *dI/dV* stabilization and feedback opening in *dI/dV* maps is 0.5 V and 200 pA. Lock-in modulation is 1 mV r.m.s. for the *dI/dV* spectrum and 4 mV r.m.s. for the maps. **(b,c)** Selected high resolution spectra of QWn resonances and accompanying vibrations FC satellites for different energies, GNR lengths and singly/doubly occupancy of frontier states. The peak corresponding to the molecular state, and replicas R_1 and R_2 are indicated by dotted lines. The abscissae are the energy difference with respect to the corresponding peak energy of each QWn state. Note that (c) shows occupied states and so the energies are negative, whereas (d) shows unoccupied states. Singly occupied/unoccupied molecular states are represented by down/up arrows. In order to facilitate the comparison with the theoretical panel the main molecular state is shaded. $\omega_{1,2}$ stand for the energy of the most intense vibrational modes (R1,2 in panel a) obtained as the average value extracted from 14 different spectra for each bias sign with different QW order (n) and GNR legth (L). Lock-in modulation: 0.2 mV rms in the spectra of *L*=11, 8 and 5-QW3↓, 1 mV rms for the rest.

Upon insertion of the GNRs onto the MgO, we observe a clear trend of the QW states to shift to lower energies. This is well understood if we look at the variation of the local work function difference ($\delta\Phi_{[Ag\text{-}MgO]}$ in the main text) between the bare Ag(001) and the embedded MgO_{ML}/Ag(001). To characterize this difference, we acquired field emission

resonances inside and outside of the MgO island, shown in Supplementary Fig. 7a. We observe a marked decrease in the bias difference between consecutive resonances (Supplementary Fig. 7b) in MgO, characteristic of a much lower workfunction. Applying a parallel plate capacitor model for the tip-sample gap [5,6], we can use the higher order resonances to give an accurate estimate of $\delta\Phi$=0.63±0.12 eV following the analysis shown in Supp. Fig. 7d.

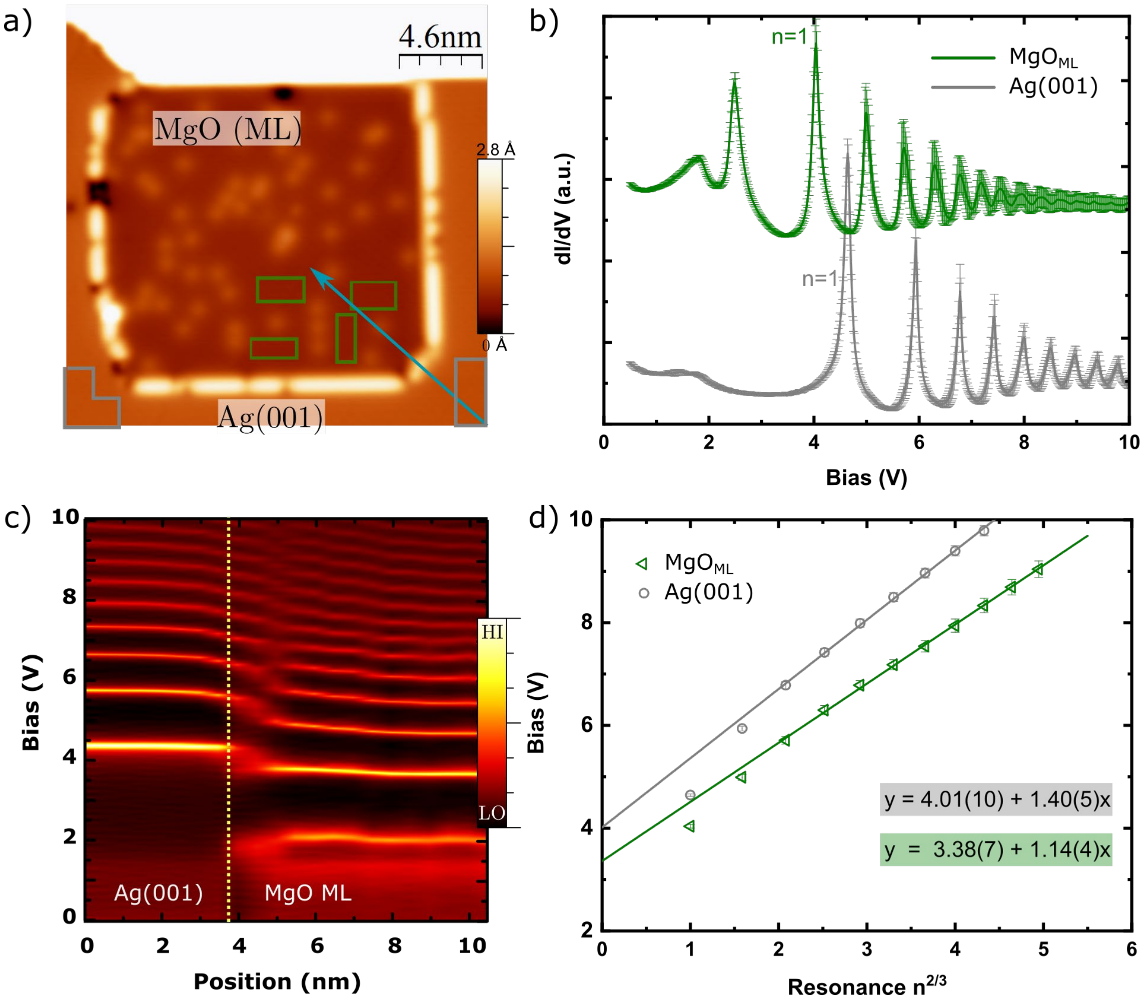

Supplementary Figure 7.- Measuring the workfunction of MgO/Ag(001). a) STM image of a MgO_{ML} island embedded in Ag(001) (0.5V and I_t=100 pA). b) Field emission resonance (FER, stabilization at 0.5 V and 100 pA) measurements recorded on Ag(001) and MgO_{ML} inside the regions enclosed by the rectangles. Solid lines are mean values and error bars are the standard deviation of a set of 90 and 66 curves for Ag and MgO respectively. c) Stack plot of *dI/dV* spectra recorded along the blue arrow in a). The dotted yellow line corresponds to the Ag-MgO intersection in STM images. d) Peak voltages of FER shown in b) plotted against the resonance order to the power of 2/3. The intersect of the linear fits with the ordinate axis gives a very precise estimate of the work function values (the two first FERs are disregarded because in this regime the 1D approximation for the electric field leading to the model in Refs. [5,6] fails). Error bars (smaller than the dot size) are calculated as the maximum range of each peak energy distribution. Statistical errors in the linear fit to the model are obtained as the largest difference between parameters derived for the linear fits of the mean peak values, the mean values with maximal positive, and the mean values with maximal negative errors. Note the peaks below 3 V are features of the Ag an MgO electronic structure, and they are not taken into account for the analysis of the workfunction.

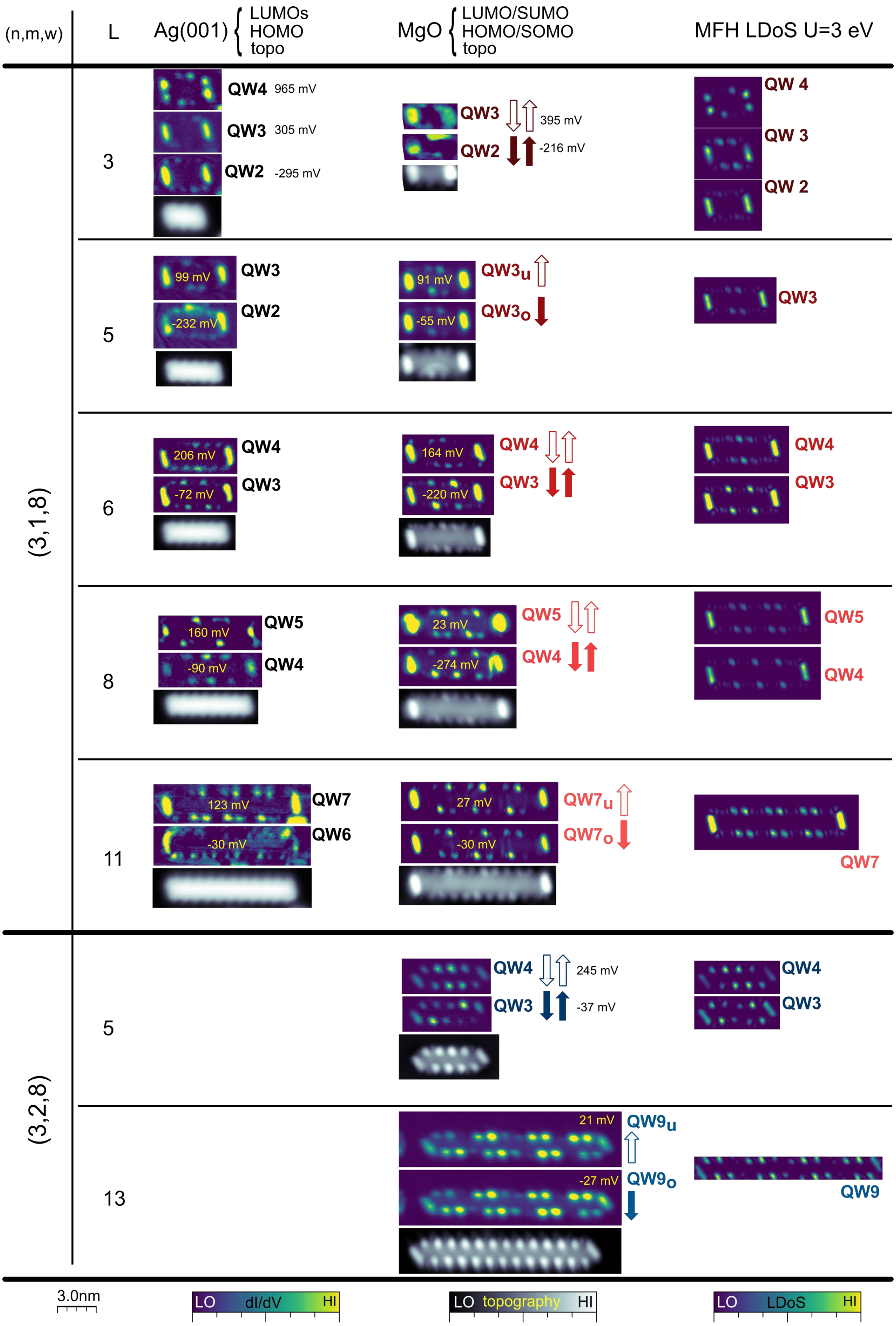

Supplementary Figure 8.- Collection of experimentally mapped frontier states in Ag(001) and MgO. Experimental constant height *dI/dV* maps are compared to theoretical simulations using the MFH model, both represented in viridis color scale adjusting the contrast to facilitate the identification of QW states. Representative STM topographies are given in bone color scale. When the gap in MgO is much smaller than in Ag(001), the two frontier states have the same spatial distribution and thus the

ground state becomes spin ½. The comparison with the calculated LDoS QW states with the same spatial distribution allows us to determine the GNRs charge state for each case. STM parameters for GNRs on MgO: constant height scans at the specified sample bias with lock-in modulation of 2 mV rms, except for L=3 for which we plot a constant current scan with regulation set point of 500 mV and 20 pA and modulation 4.4 mV rms. STM parameters for GNRs on Ag(001): constant current maps at the specified sample bias for L=3, 5, 6, 11; constant current maps with regulation set point of -300 mV and 100 pA for L=8. Scale bar in the bottom applies to all images. All topographies (grey scale images) are taken at 500 mV sample bias. Labels and color code of schematic spin states are the same as in Figs. 3 and 4 of the main text.

(n,m,w)	L / pu	Ag(001)			MgO_{ML}/Ag(001)			$\Delta E_{MgO\text{-}Ag}$
		Last occ. QW (k^{th})	INT(#e)	e/PU (≃)	Last occ. QW (k^{th})	#e	e/PU	Shift $(k+1)^{th}$ QW / eV
318	3	2	6	2	2	6	2	+0.075
	5	2	6	1.2	**½ 3**	7	1.4	-0.1
	6	3	8	1.33	3	8	1.33	-0.07
	8	4	10	1.25	4	10	1.25	-0.137
	10	4	12	1.2	--	--	--	--
	11	6	14	1.27	**½ 7**	15	1.36	-0.123
	12	5	14	1.16	--	--	--	
328	5				3	8	1.6	
	13	--	--	--	**½ 9**	19	1.46	

Supplementary Table 1.- Experimental charge states of chiral GNRs. List of approximate charge states on Ag(001), integer charge states on MgO_{ML} last occupied QW states and shift of the first fully unoccupied QW state. The order of the QW state has been deduced by comparing experimental dI/dV maps of the frontier orbitals with the theoretical LDoS. The ½ symbol indicates that the referred QW state is singly occupied.

Figure 85 collects the experimental LDoS maps of the QW resonances for all the GNRs that were successfully characterized on the MgO_{ML}, together with the corresponding states as they appear on the bare Ag(001). A comparison with the simulated LDoS using the MFH model with U=3 eV (see Methods at the main article body), shown in the right hand column, allows us to extract the quantum number associated to the QW order, and thus, the charge state (as explained at the discussion of Fig. 3 of the main text). The so deduced charge states and resulting doping densities for GNRs on Ag and MgO_{ML} are given in Table S1.

The first unoccupied QW state experiences a shift in energy of approximately -100 meV when moving the GNR (L>3 PU) from the Ag to the MgO_{ML} (schematically represented by grey dashed lines in Fig. 3 of the main manuscript). Surprisingly, this shift and the concomitant charge transfer from the underlying metal, occurs in a step-wise manner as more PU units are positioned over the MgO during the insertion. This is shown in Supplementary Fig. 9, where we provide *dI/dV* spectra of 4 different GNRs in different stages of the lateral atomic manipulation process.

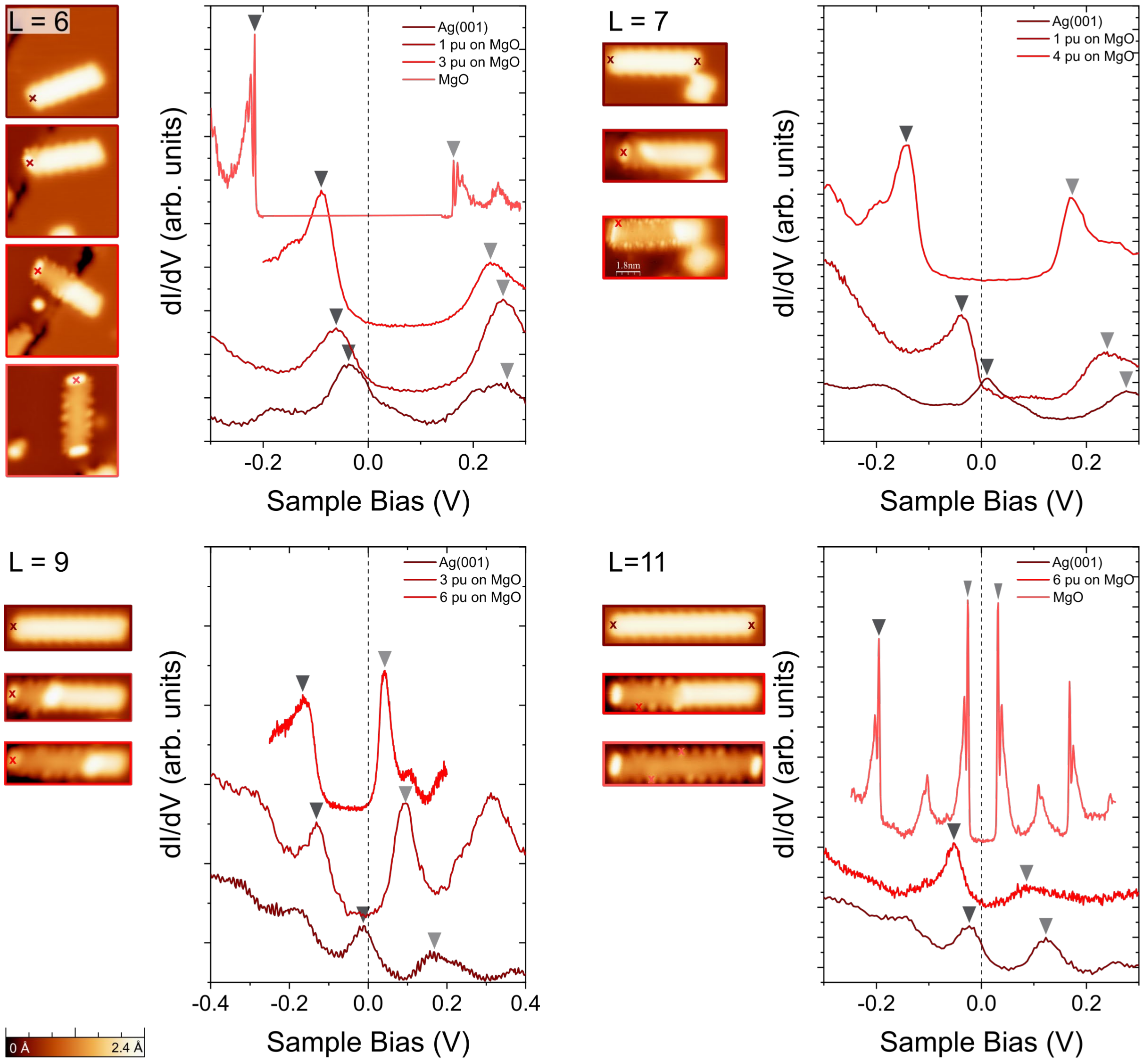

Supplementary Figure 9.- Experimental demonstration of electron doping of GNRs entering MgO. The insets illustrate the gradual insertion of (3,1,8)-GNRs on MgO and concomitant rigid downshift of the QW states. Horizontal image size: *L*=6 → 8 nm; *L*=7→ 9 nm; *L*=9→9 nm ; *L*=11→11.5 nm. The correlations induced splitting of the edge state only appears for fully decoupled ribbons. When some portion of the GNR remains in contact with the Ag(001), the n-doping takes place gradually depending on the length inserted onto MgO_{ML} and metal, but the e-e interaction is partially quenched. This explains why previous attempts with (1,0,6)-GNR/Au(111) partially intercalated with NaCl [7] did not manifest the true discretization observed on our MgO_{ML} patches.

III. Supplementary Note 3.

In this Supplementary Note we discuss the identification of the SPT state and the charge neutrality point of GNRs. The charge neutral GNRs are characterized by a half-filled SPT state [8], and therefore the binding energy of this state will give the charge neutrality energy level for the charged case. The simulated DOS of a 11PU (3,1,8)-GNR with the observed charge state (15 e-) is shown in Supplementary Fig. 11). The QW resonances with low index (close to the SPT states) are very close in energy (they correspond to the first LUMOs of the neutral GNR, which originate from discretization of the parabolic conduction band of the edge states), and merge into a broad peak as a consequence of the overlap with vibrational resonances and the small intrinsic linewidth of all the spectroscopic features. This broad band is peaked at the value of the SPT end states, which lie at -410 meV in the simulated DOS and -650 meV in the experimental dI/dV spectra. The downshift of the SPT end states is due to the 500 meV change of the chemical potential, which is the value yielded by our calculation -based on Supplementary Eq. (2)- to fill the the GNR with 15 electrons.

The experimental part of Supplementary Fig. 11 shows a dI/dV spectrum in a wide bias region, showing a set of multiple peak features. Through the analysis of the dI/dV maps and comparing with the corresponding ones in MFH simulations, we can safely identify the half occupation of QW7 and states around this orbital (QW6-QW8). We note that while these peaks may change their intensity on different GNR positions, they are always detected at the same energy position. The peaks are very sharp and, therefore, very sensitive to the variations of the electrostatic potential at MgO surface (e.g. see the parabolic shape of the line spectra in Fig. 2 of the manuscript). This causes small discrepancies in the intensity of the maps with respect to DOS maps, which can only be corrected applying the FD-STS method explained in Supplementary Section V and Supplementary Fig. 14a-b. However, the number of nodal planes allows us the right identification of each QW state.

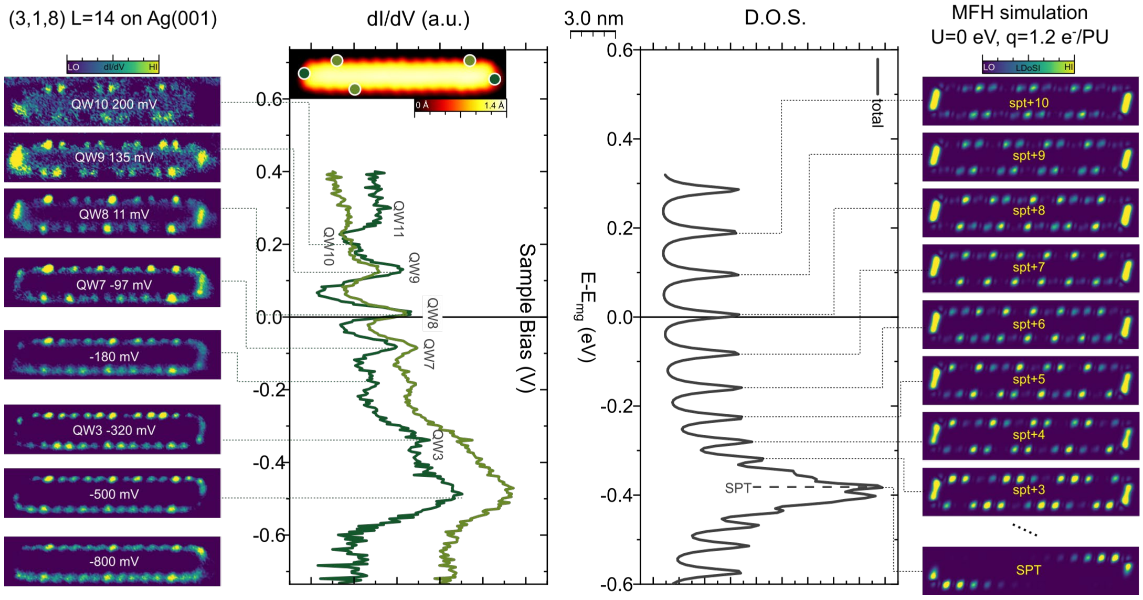

Supplementary Fig. 10.- Electronic structure of GNRs on Ag(001) in a broad bias range. From left to right, constant current dI/dV maps of several quantum-well (QW) states of the 14 P.U. (3,1,8)-GNR on Ag(001), corresponding dI/dV spectra, calculated total density of states (DOS) within MFH model (see Methods) and the corresponding simulation of the spatial distribution of each eigenstate. Experimental dI/dV curves are averaged individual spectra taken in the positions marked by the dots

with the same colour code. STM parameters for maps and spectra: regulation set point 0.5 V/200 pA, V_{mod}=5 mV. The broad peak found at around -0.5 V can be explained as the overlap of several low order QW resonances next to the SPT state of the charge neutral GNR. MFH simulations are obtained for a negative charge excess equivalent to 16.8 electrons and U=0 eV. DOS is schematically represented by the eigenenergies with a Lorentzian broadening of 20 meV, and simulations of the DOS maps are taken at a height of 1.8 nm. Black scale bar applies to all images.

Below these energy (and, also, above), sharp dI/dV peaks (marked with asterisks in Supplementary Fig. 11) changing abruptly its energy with the tip position are attributed to charging events mediated by point defects of the MgO substrate (see Supplementary Fig. 14e-k). Most probably, that peak is also contributed by QW4 and the vibrational resonances of QW5, which can be also shifted differently in each charge state of the defect. Consequently, the dI/dV maps in this energy range of -400 to -600 meV show features that cannot be uniquely assigned to a single QW state, although the dI/dV intensity pattern of at -500 meV is very close to that of the simulated QW3, and the characteristic intensity at the acute corner of the ribbon in SPT states is visible in the -662 mV map.

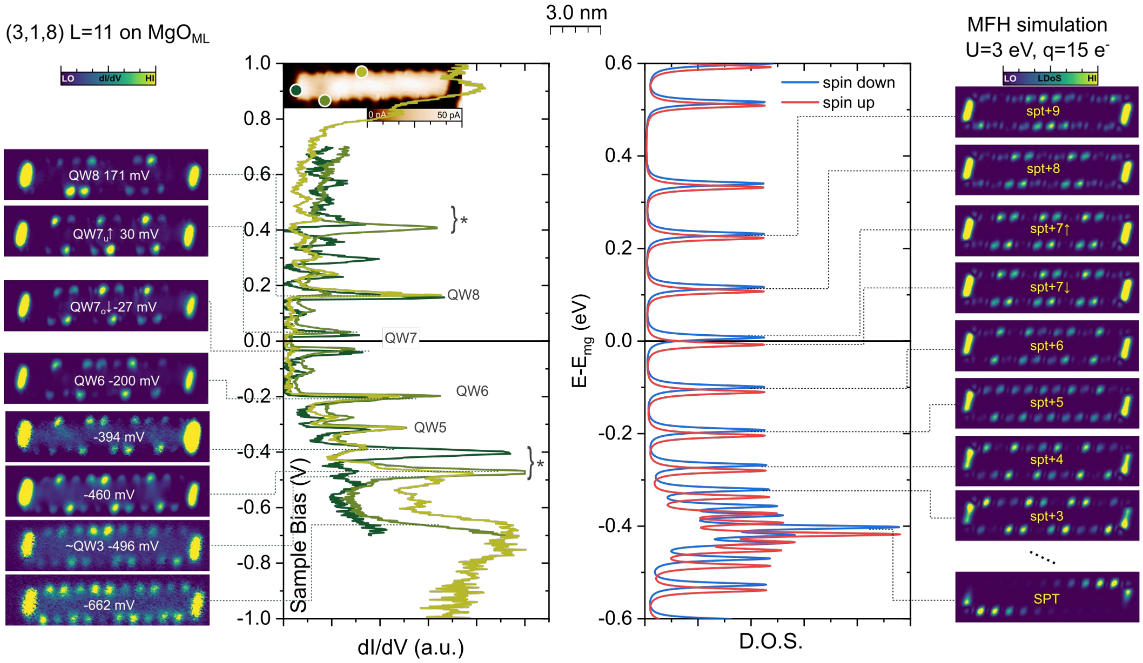

Supplementary Fig. 11.- Electronic structure of GNRs on MgO/Ag(001) in a broad bias range. From left to right, constant height dI/dV maps of several quantum-well (QW) states of the 11 P.U. (3,1,8)-GNR on MgO, corresponding dI/dV spectra, calculated total density of states (DOS) within MFH model (see Methods) and theoretical simulation of the spatial distribution of each eigenstate. Experimental dI/dV spectra are taken in the positions marked by the dots with the same colour code at the inset showing the constant height current map. STM parameters for maps: regulation set point at ribbon centre is 50 mV/20[100/5] pA for QW8[QW7/QW6] and 0.5V/200 pA for the rest, V_{mod}=1 mV. STM parameters for spectra: stabilization at -0.5 V/200 pA, V_{mod}=2 mV. The images of occupied/unoccupied spin split QW7 states are labelled by down/up arrows. The charging peaks are indicated by an asterisk (*). The broad peak found at around -0.65 eV can be explained as the overlap of several low order QW resonances next to the SPT state of the charge neutral GNR. MFH simulations are obtained for 15 electrons excess and U=3 eV. DOS is schematically represented by the eigenenergies with a Lorentzian broadening of 2 meV, and simulations of the DOS maps are taken at a height of 1.8 nm. Black scale bar applies to all images.

The charge neutrality point can be probed at this large negative bias: we observe an increase of dI/dV attributed to the broad spectral band predicted by MFH simulations. According to the above discussion, this band, peaked around -0.65 eV, is the indication of

the neutrality point of the GNR. This state appears ~200 meV lower than predicted by the model. This is probably due by the combined action of downwards shifts caused by i) the charging events, ii) a small potential drop that may occur across the MgO layer, and iii) some renormalization of levels in the charged molecule. Furthermore, our MFH model ubiquitously uses 3 eV as on-site Coulomb repulsion energy. Fine tuning of this parameter would allow us to adjust the chemical potential in equilibrium. Still, the simulated long-range spectrum is consistent with our identification of frontier orbitals, and our parametrization captures all essential Physics of the system.

Supplementary Fig. 10 includes a set of similar maps and spectra for a 14PU (3,1,8)-GNR on Ag(100). In this case, QW8 and QW9 are clearly identified as Frontier states, consistent with an electron charge between 16-18 electrons. Other QW states (e.g. QW10 and QW3) can also be identified from the map's comparison as no charging events take place on the metal. Regarding identification of lower QW states and the neutrality point, we note that on the Ag(100) surface the QW peaks are wider. The lower QW states merge in a broad state peaked at 500 meV below zero, which is pictured in the MFH simulations as the position of the SPT state of the charge neutral case. This value is also in line with the predicted shift of our model for a charge transfer of 1.2 e^-/PU of about -400 meV.

IV. Supplementary Note 4.

In this supplementary section we provide a detailed description of the charging model within the grand canonical ensemble formalism.

It is useful to describe the Ag(100)/MgO_{ML}/GNR system of our experiment as a plate capacitor model formed by components with different electron affinity (E_a). This model is validated in Ref. [9]. The MgO layer acts as electronic decoupler, that hinders the wavefunction overlap between metal and molecular states, and stabilizes integer states in the GNR (rather than fractional, as would happen for direct GNR adsorption on the metal). At the same time, the MgO layer reduces the substrate's work function [10–12]. To reach chemical equilibrium with the metal, the GNR becomes charged by an integer amount of electrons that tunnel from the metal through the MgO layer in response to their difference in electron affinities. The charging of the GNR simultaneously causes an electric field across the MgO that opposes the charging and partially compensates the difference in electron affinities. This component has been described in previous works as an interface dipole that causes deviation from pure vacuum-level alignment [9]. Therefore, in equilibrium these processes are reflected by a shift of the GNR's chemical potential μ with respect to the neutral case level μ_0 (i.e. by $\Delta\mu = \mu - \mu_0$), which is smaller than the difference in electron affinities owing to the energy U_d stored in the MgO capacitor by the built-up dipole. This is reflected in the following expression

$$\Delta\mu = \mu - \mu_0 = E_a - \Phi - U_d \quad (1)$$

The value of $\Delta\mu$ determines the charge state of a GNR of length L in equilibrium. The two types of chiral GNRs used in the experiment have a non-trivial topological band structure and, therefore, two symmetry-protected topological (SPT) end states [8]. For the neutral GNR, the two SPT states are half occupied, and therefore the neutral level μ_0 is referenced by their binding energy (Fig. 5a main article). Upon electron charging, the chemical potential μ will lie either between two LUMO levels of the molecule for even charge (i.e. the closed-

shell configuration, singlet spin state), or between SOMO and SUMO levels, for the case of odd charge state (open shell, doublet spin state).

To simulate how the GNR charges as a function of length, we describe the system in the grand canonical ensemble and calculate the mean number of excess electrons $\langle q \rangle$ for any given chemical potential μ using the relation

$$\langle q(\mu, L, T) \rangle = -k_B T \frac{d\mathcal{Z}(\mu,L,T)}{d\mu} \qquad (2)$$

This expression provides a charge value as a function of μ which can be compared with experimental results for every GNR. For a GNR with a given length L and chirality, at the temperature of our experiment (between 1.2 and 4.3 K), we can calculate the grand canonical partition function $\mathcal{Z}(\mu, L, T)$ following the expression

$$\mathcal{Z}(\mu, L, T) = \sum_j exp\left(\frac{N_j T - E_q}{k_B T}\right) = \sum_q exp\left(\frac{j\mu - E_0 - E_q}{k_B T}\right) \quad (3)$$

where N_j is the total number of indistinguishable electrons, and E_j the internal energy of each respective charge state j obtained from MFH simulations (see Methods main text). No overcounting correction is included as we consider each microstate j only once. We note that at cryogenic temperatures, the entropic contribution to the grand potential can be neglected and $\langle q \rangle$ can be obtained equivalently by simply minimizing the right hand side of Supplementary Eq. (3), instead of the total energy. We limit our model to the spin-restricted case, as the mean spin densities on the edge are energetically unstable against the emergence of nodal planes, and thus determining the correct ground state becomes ambiguous for large (3,*n*,8)-GNRs doped into the chiral edge band.

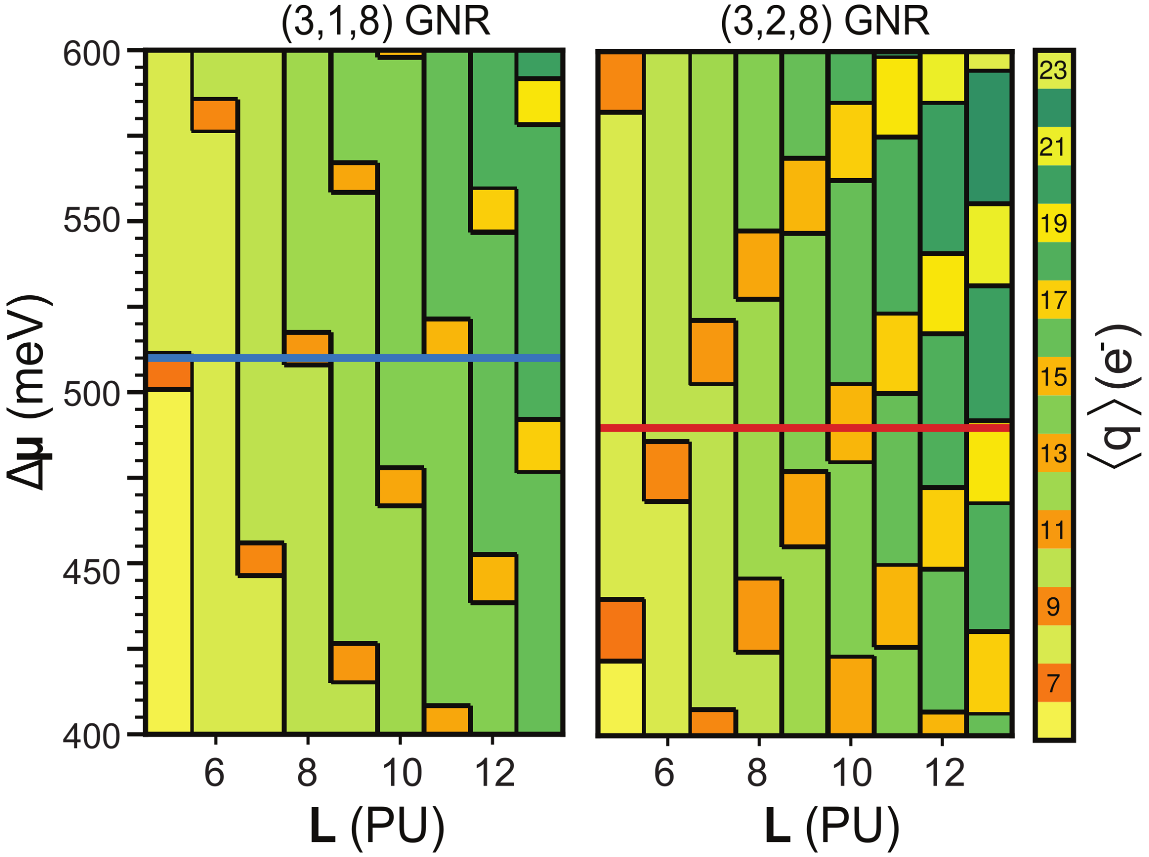

Supplementary Fig. 12.- Calculated charge excess in chiral GNRs. Contour plots of the calculated excess charge for (3,1,8)- (left) and (3,2,8)-GNRs (right). Even and odd charge

states are represented by different colour scales. Horizontal lines represent the best-fit values for $\Delta\mu$ to match the experimental charging pattern (see Fig. 5b of the main text).

Supplementary Fig. 12 provides the predicted charge patterns as a function of GNR's length as the chemical potential shifts $\Delta\mu$ from 400 to 600 meV. The map reflects the increase in charge with length for both chiralities, and the peculiar odd-even (orange-green) charge pattern that depends on the value of $\Delta\mu$. Comparing the charging patterns with the experimental charge states of the GNRs (Fig. 4 of the main text), an estimated value for $\Delta\mu$, the chemical potential shift, is determined and represented in Supplementary Fig. 12 by horizontal lines. The theoretical pattern and its comparison with experiments is plotted in Fig. 5b of the main text. The only deviation occurs for the ribbon (3,1,8)-GNR with L=8, which appears with even electron occupation (q=10) in the experiment, while in the map is expected to have 11 elementary charges. As discussed in Section V, small variation of $\Delta\mu$ caused by local defects in the MgO layer can explain this discrepancy.

V. Supplementary Note 5.

In this supplementary section we report our results for the model of vibrational modes of finiete chiral GNRs on MgO. We performed calculations of the vibrational modes of chiral GNRs using atomistic model potentials with the GULP package (see Gale and Rohl, Molecular Simulation 29, 291 (2003)) [13]. In detail, we modelled the GNR itself by an accurate potential for hydrocarbons [14] while the molecule-surface interaction is described by simple Lennard–Jones 12-6 potentials between pairs of atoms with a varying strength E_{LJ}. We treat the MgO film as a rigid surface.

The total density of vibrational modes (each one represented with smearing factor of 3 meV) is shown in the negative scale of Supplementary Fig. 13 for different number of precursor units (L) and interaction strength with the model substrate (E_{LJ}). To nail down the origin of these modes, we computed the out-of-plane density of vibrational states as the norm of the z-components of their displacement eigenstates, which is shown in the positive scale of the figure.

The first peak in the spectrum at lowest energy corresponds to an out-of plane translation of the molecule's center of mass (COM), thereby an external vibration. Its energy scales with E_{LJ}, and we therefore use this parameter to match the observed mode energy at 7.5 meV (see experimental vibrational resonances in Supplementary Figs. 6b-c). This indicates that the concerted movement of the whole GNR with respect the MgO surface is the most probable mode describing the 7.5 meV FC resonance. In the out-of-plane part of the vibrational spectrum, a secondary band emerges in the energy range 70-130 meV. These vibrational modes include those with predominantly H-atoms moving out-of-plane. The onset of this secondary band is very similar to the 76 meV peak observed in the experiments (see Supp. Fig. 6). As shown in Supplementary Fig. 13, these features are rather insensitive to the length of the GNR, in agreement with the experimental data.

At higher energies there are additional vibrational modes in the region corresponding to the C-C stretching modes, which disappear when only vertical motion is considered (because they do not have out of plane components). As shown in Supplementary Fig. 13, these internal modes do not react to the GNR-MgO interaction potential strength, whereas the external modes are clearly affected, outlining their different nature.

In summary, this simple analysis suggests that the two satellite peaks experimentally observed in dI/dV may be ascribed to the excitation of out-of-plane vibrational modes, and more specifically to the external out-of-plane COM vibration and to the out-of-plane H-atom motion, respectively.

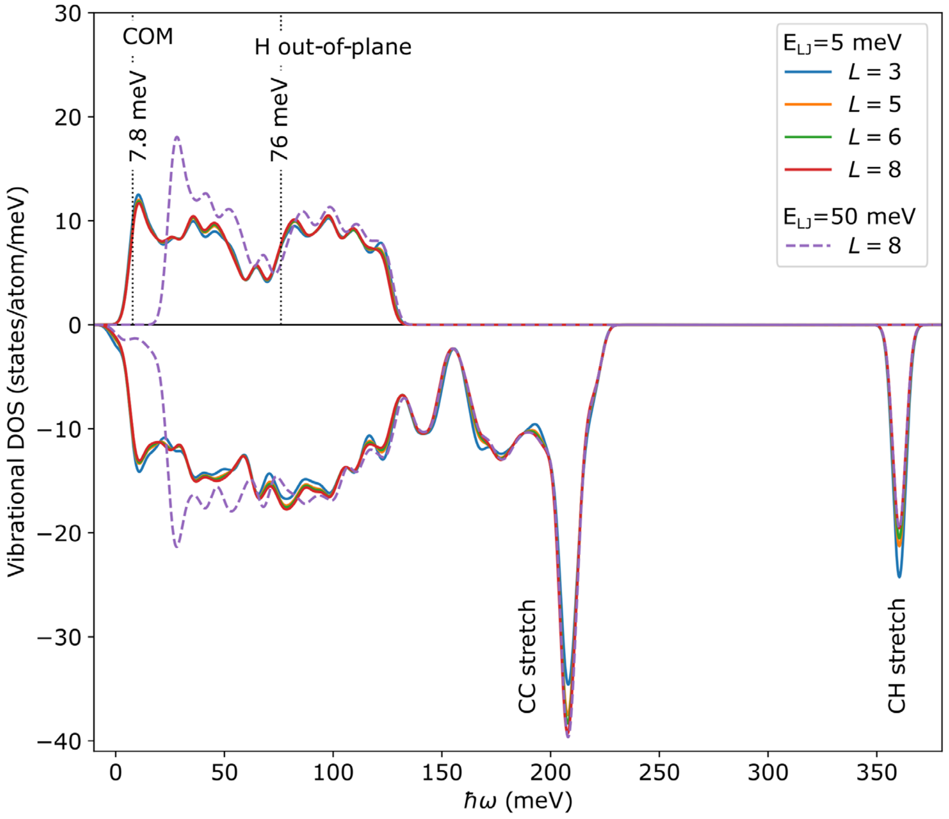

Supplementary Fig. 13.- Theoretical vibrational density of states for (3, 1, 8)-GNRs. We model the role of the MgO film by an inter-atomic interaction described by a 12-6 Lennard-Johns potential with strength given by the energy scale of E_{LJ}. The negative DOS corresponds to the total vibrational states. The positive DOS represent out-of-plane vibrations. The discrete spectrum is broadened by a Gaussian function with a smearing of 3 meV. Dotted lines mark the energy of the experimentally obtained most intense vibrational peaks (see Supplementary Fig. 6), which are ascribed to vertical vibrations of the GNR center of mass (COM, thereby the ribbon as a whole) and to the concerted out-of-plane vibration of the hydrogen atoms.

VI. Supplementary Note 6.

In this Supplementary Note we provide further evidence of electrostatic gating of chiral graphene nanoribbons on MgO. As shown in Supplementary Fig. 14a-c and Fig. 2 of the main article, when the tip moves laterally along the GNRs on MgO, we often observe a rigid shift of all energy levels, ΔE_p (which would not be visible in the case of molecular orbitals spanning in an energy range wider than the maximum ΔE_p~20 meV). In several cases we have been able to find a direct relationship between the ΔE_p and the lateral tip distance to point defects of the MgO that can be detected either below the GNR or next to it. In Supp. Fig. 14 we present an example of this effect for the (3,1,8)-GNR with L=11 discussed in the main text.

If we apply the Feature Detection STS method [15] to compose a map of the area where a spectroscopic feature with the shape of the QW resonances (i.e., a peak with FWHM of 1.3 mV, see Supp. Fig. 5) exists, the intensity pattern of the QW edges is evenly distributed at the locations predicted by MFH simulations (Supplementary Fig. 14d). This is in contrast with the case of constant height dI/dV maps at a given energy of the same GNRs, where some lobes of the QW state appear brighter than others (see for example Supp. Fig. 8 or Fig. 3b). The reason is that as the tip position $\vec{\boldsymbol{r}} = (x, y, z)$ departs from the defect at $\vec{\boldsymbol{r}}_0 = (x_0, y_0, z_0)$, the electrostatic potential energy at the defect $U_e(\vec{\boldsymbol{r}} - \vec{\boldsymbol{r}}_0)$ varies. Indeed, it has been previously shown that the electric field in the tip-sample gap can modify controllably the electric polarization of thin insulating layers [16], specially next to point defects and vacancies. This gives rise to a variation of the local value of $\Delta\mu(\vec{\boldsymbol{r}} - \vec{\boldsymbol{r}}_0)$ which, in the parallel plane approximation, will be proportional to U_e. This effect can be viewed as an effective gating of the molecular states of the GNRs.

The location of the defect can be pulled out from measurements of the tip induced charging resonances of the defect states [4,17]. They manifest as sharp ellipsoidal rings in the (x, y) plane enclosing the region for which $U_e(\vec{\boldsymbol{r}} - \vec{\boldsymbol{r}}_0)$ is large enough as to charge (positively or negatively) the defect at a constant $z - z_0$. Fixing V_b at a large value of 0.5 V, the size of the charging ring must depend linearly on the tip sample distance, or equivalently, logarithmically on the current set point I_t before opening the feedback (see Supplementary Figs. 14e-k). As shown in Supplementary Fig. 14j, we can determine accurately the defect position as the centre of an ellipse fitting the charging ring for different tip heights.

Alternatively, the defects can be found in atomically resolved images of the MgO patch after removing the GNR under study, as illustrated in Supplementary Fig. 15. Here, we show how the QW states of the (3,1,8)-cGNR with L=5 PU can be shifted in energy by changing the distance to a point defect on MgO_{ML}. The GNR in position γ lies over a defect free region of the MgO, and displays a correlation gap between singly occupied frontier states –as predicted by the model discussed in supplementary theoretical methods-. In contrast, when the GNR lies over a point defect (position α) or next to the MgO bilayer edge (position β), the 3rd QW state (see Supplementary Fig. 8) is shifted in energy above the Fermi level and becomes fully unoccupied, yielding another charge (q=6 electrons instead of 7) and total spin (S=0 instead of S=1/2).

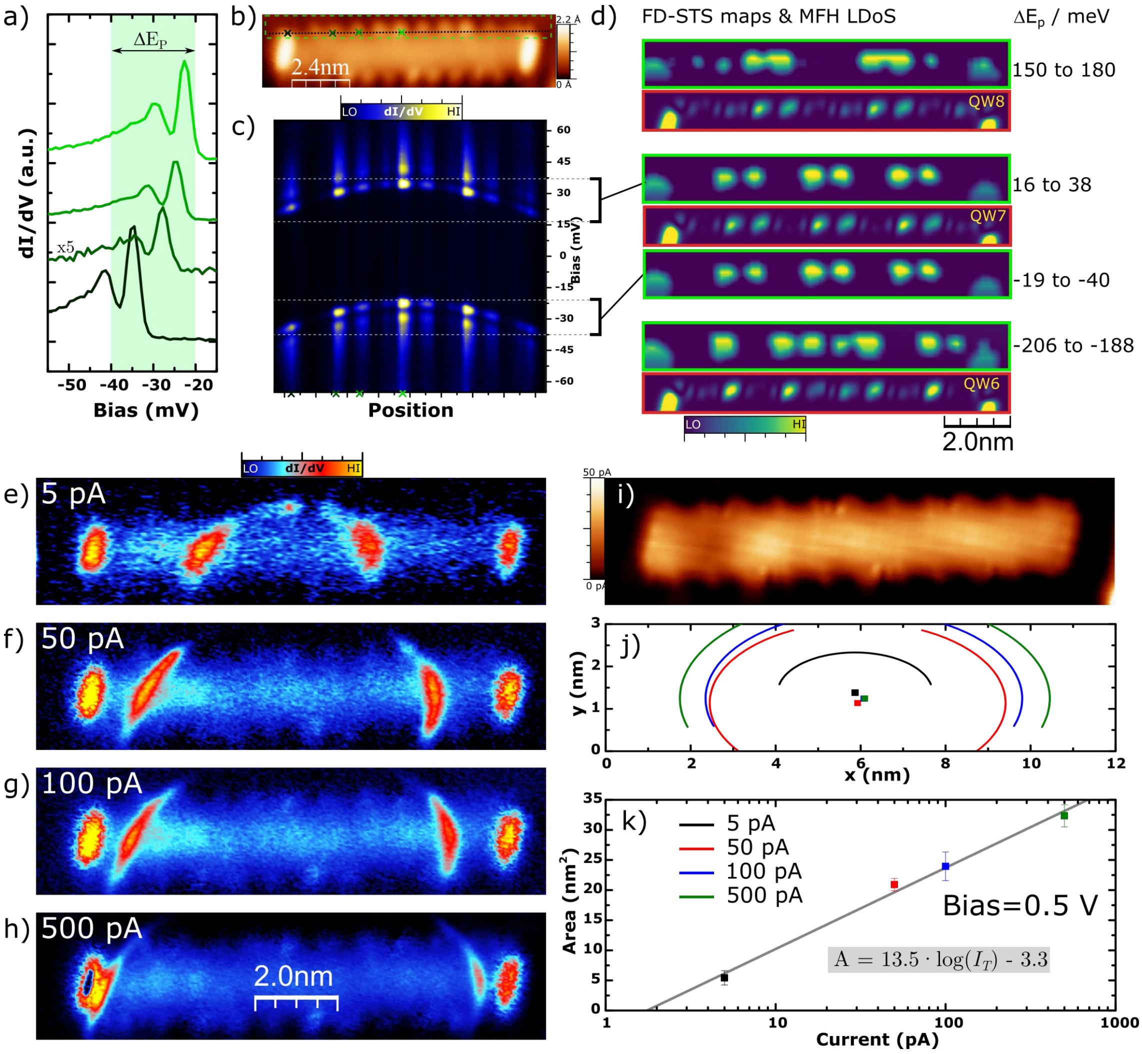

Supplementary Figure 14.- Gating energy levels by lateral tip positioning. (a-c): Shift in energy along the chiral edge of the (3,1,8)-GNR with L=11. (STS set point: 0.5 V, 200 pA, V_{mod}=1 mV). a) STS spectra recorded at different positions of the chiral edge. b) STM topography image of the L = 11 (3,1,8)-GNR including crosses where STS spectra shown in (a) are recorded. Image set point: 0.5 V, 100 pA. c) Stack plot of *dI/dV* versus position recorded on the chiral edge of the GNR shown in b (dotted line). d) Feature-Detection STS [15] intensity maps tuned to a peak with linewidth ≤2 mV in the energy range ΔE_p indicated on the right column. Due to the high spatial and energy resolution required for this analysis, we only studied the frame enclosed by the dashed green rectangle in (b). Each experimental map is compared with the corresponding theoretical LDoS of the QW states obtained from the MFH model with U=3 eV and q=15 electrons (see theoretical methods at the main text) for the L = 11 (3,1,8)-GNR. e-k) Determination of the location of point defects in MgO_{ML} by high bias mapping. e-h) Series of constant height *dI/dV* maps recorded at 0.5 V with tunnelling current set points from 5 to 500 pA at the time of feedback opening over the ribbon centre (V_{mod} = 4 mV). i) In-gap constant height STM image of the GNR shown in e-h (V_b=-2.5 mV). j) Fits (lines) and centers (squares) of the ellipses extracted from the *dI/dV* maps. k) Area of the ellipses as a function of set point tunnelling current. The error bars are computed from the standard deviations of the ellipse's major and minor axes yielded by the fit.

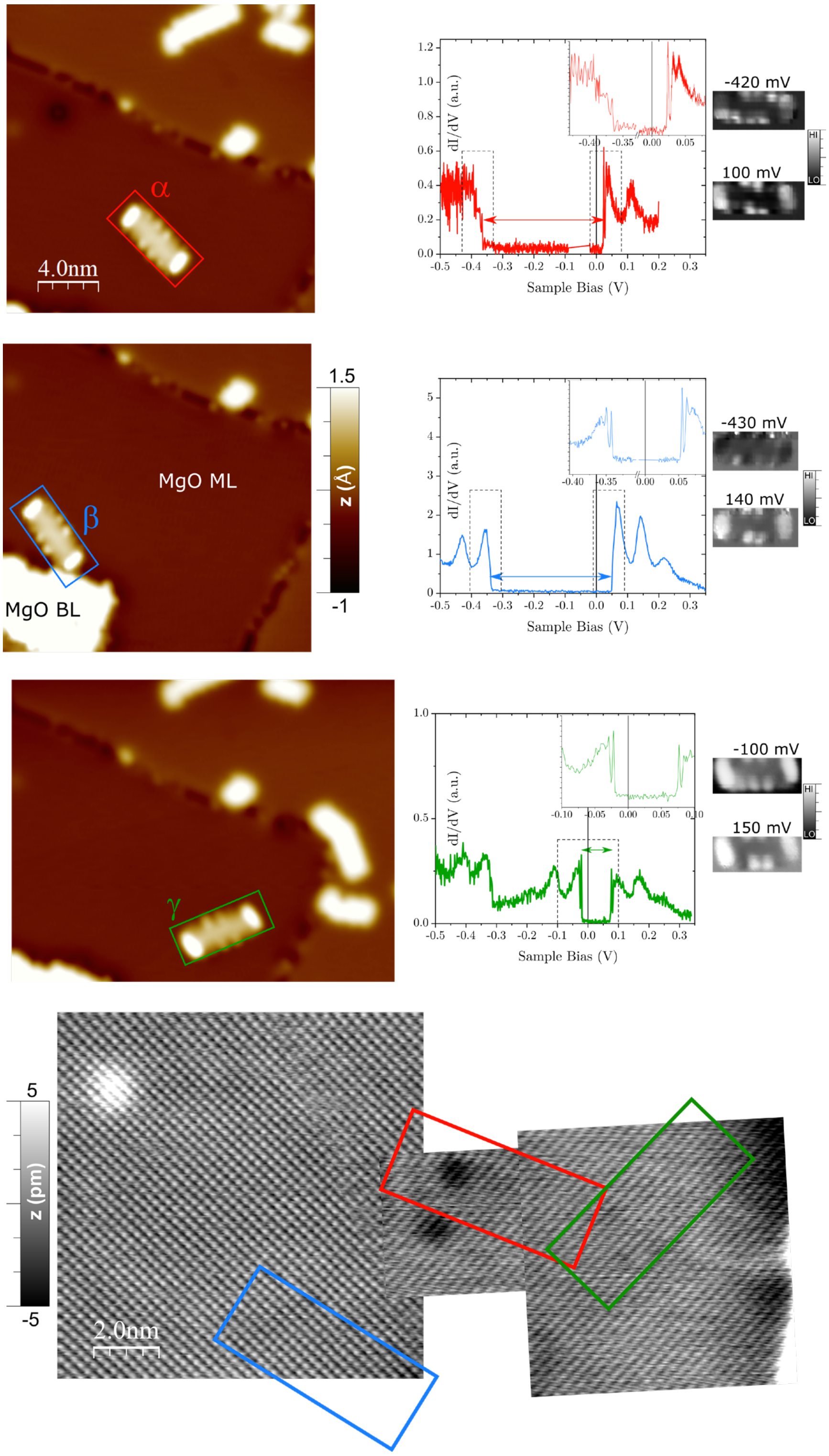

Supplementary Figure 15.- Gating electron occupancy by GNR position. Data corresponds to the L=5 (3,1,8)-GNR. V_{mod}=1 mV and 0.5 mV r.m.s. for spectra in main panels and in the insets, respectively (except for the β position, for which the main panel spectra is taken with V_{mod}=5 mV). The maps of the QW states are tunnelling current images taken at the referred energies but regulating at each pixel at 0.5 V. The three upper panels show the same GNR at three different positions over the MgO patch (colour scale applies to all of them). As shown in the atomically resolved image of the substrate (grey color scale), in position α the GNR is over a dark spot and next to the bright defect on MgO. It features a normal quantization gap and q=6 electrons and therefore total spin S=0. Position β is next to the MgO bilayer (BL) island, and the GNR has same charge/spin state as in α. However, at the γ

position, farther away from all types of defects, the GNR is over a defect free region of the MgO, and now exhibits a much smaller gap, as corresponds to the correlations gap. In this latter case, the current map integrating the resonance of the frontier states are the same at both sides of the Fermi level, and thus it has q=7 electrons (odd occupancy) and total spin S=1/2.

VII. Supplementary References

[1] A. Berdonces-Layunta, F. Schulz, F. Aguilar-Galindo, J. Lawrence, M. S. G. Mohammed, M. Muntwiler, J. Lobo-Checa, P. Liljeroth, and D. G. de Oteyza, Order from a Mess: The Growth of 5-Armchair Graphene Nanoribbons, ACS Nano **15**, 16552 (2021).

[2] G. Reecht, N. Krane, C. Lotze, L. Zhang, A. L. Briseno, and K. J. Franke, Vibrational Excitation Mechanism in Tunneling Spectroscopy beyond the Franck-Condon Model, Phys. Rev. Lett. **124**, 116804 (2020).

[3] J. van der Lit, M. P. Boneschanscher, D. Vanmaekelbergh, M. Ijäs, A. Uppstu, M. Ervasti, A. Harju, P. Liljeroth, and I. Swart, Suppression of electron–vibron coupling in graphene nanoribbons contacted via a single atom, Nat Commun **4**, 2023 (2013).

[4] M. S. G. Mohammed, L. Colazzo, R. Robles, R. Dorel, A. M. Echavarren, N. Lorente, and D. G. de Oteyza, Electronic decoupling of polyacenes from the underlying metal substrate by sp3 carbon atoms, Commun Phys **3**, 159 (2020).

[5] J. Bono and R. H. Good, Conductance Oscillations in Scanning Tunneling Microscopy as a Prob of the Surface Potential, Surface Science **188**, 153 (1987).

[6] O. Yu. Kolesnychenko, Yu. A. Kolesnichenko, O. I. Shklyarevskii, and H. van Kempen, Field-emission resonance measurements with mechanically controlled break junctions, Physica B: Condensed Matter **291**, 246 (2000).

[7] S. Wang, L. Talirz, C. A. Pignedoli, X. Feng, K. Müllen, R. Fasel, and P. Ruffieux, Giant edge state splitting at atomically precise graphene zigzag edges, Nature Communications **7**, 11507 (2016).

[8] J. Li, S. Sanz, N. Merino-Díez, M. Vilas-Varela, A. Garcia-Lekue, M. Corso, D. G. de Oteyza, T. Frederiksen, D. Peña, and J. I. Pascual, Topological phase transition in chiral graphene nanoribbons: from edge bands to end states, Nat Commun **12**, 5538 (2021).

[9] P. Hurdax, M. Hollerer, P. Puschnig, D. Lüftner, L. Egger, M. G. Ramsey, and M. Sterrer, Controlling the Charge Transfer across Thin Dielectric Interlayers, Adv. Mater. Inter. **7**, 2000592 (2020).

[10] M. Hollerer, D. Lüftner, P. Hurdax, T. Ules, S. Soubatch, F. S. Tautz, G. Koller, P. Puschnig, M. Sterrer, and M. G. Ramsey, Charge Transfer and Orbital Level Alignment at Inorganic/Organic Interfaces: The Role of Dielectric Interlayers, ACS Nano **11**, 6252 (2017).

[11] G. Witte, S. Lukas, P. S. Bagus, and C. Wöll, Vacuum level alignment at organic/metal junctions: “Cushion” effect and the interface dipole, Appl. Phys. Lett. **87**, 263502 (2005).

[12] H.-J. Freund and G. Pacchioni, Oxide ultra-thin films on metals: new materials for the design of supported metal catalysts, Chem. Soc. Rev. **37**, 2224 (2008).

[13] J. D. Gale and A. L. Rohl, The General Utility Lattice Program (GULP), Molecular Simulation **29**, 291 (2003).

[14] D. W. Brenner, O. A. Shenderova, J. A. Harrison, S. J. Stuart, B. Ni, and S. B. Sinnott, A second-generation reactive empirical bond order (REBO) potential energy expression for hydrocarbons, J. Phys.: Condens. Matter **14**, 783 (2002).

[15] J. Martinez-Castro et al., Disentangling the electronic structure of an adsorbed graphene nanoring by scanning tunneling microscopy, Commun Mater **3**, 57 (2022).

[16] J. Martinez-Castro, M. Piantek, S. Schubert, M. Persson, D. Serrate, and C. F. Hirjibehedin, Electric polarization switching in an atomically thin binary rock salt structure, Nature Nanotechnology **13**, 19 (2018).
[17] C.-L. Song, Y.-P. Jiang, Y.-L. Wang, Z. Li, L. Wang, K. He, X. Chen, X.-C. Ma, and Q.-K. Xue, Gating the charge state of single Fe dopants in the topological insulator Bi_{2}Se_{3} with a scanning tunneling microscope, Physical Review B **86**, (2012).
