# Supplementary material for: Systematic modulation of charge and spin in graphene nanoribbons on MgO
Source: Nat Commun. 2025 Jul 1;16:5632. doi: 10.1038/s41467-025-60767-5 (PMC12218530; doi:10.1038/s41467-025-60767-5)
Supplement: Supplementary file 2 — Transparent Peer Review file [file 41467_2025_60767_MOESM2_ESM.pdf]

nature portfolio

Peer Review File

Systematic Modulation of Charge and Spin in Graphene Nanoribbons on MgO

Corresponding Author: Dr David Serrate

This manuscript has been previously reviewed at another journal. This document only contains information relating to versions considered at Nature Communications.

Version 0:

Reviewer comments:

Reviewer #1

(Remarks to the Author)
The revised version of the manuscript and the reply by the authors addresses my concerns sufficiently. I recommend publication in Nature Communications.

(Remarks on code availability)

Reviewer #2

(Remarks to the Author)
Review for NCOMMS-25-14005-T
I reviewed the previous version of the manuscript. I thank the authors for their detailed response and the incorporation of all reviewers' suggestions regarding their work into the revised manuscript. The authors precisely addressed all questions, and their extensive responses clarified all the issues raised. After carefully re-visiting the content, I do not find any aspect that requires further revision, and I strongly recommend the current version for publication in Nature Communications.

(Remarks on code availability)
N/A

Open Access This Peer Review File is licensed under a Creative Commons Attribution 4.0 International License, which permits use, sharing, adaptation, distribution and reproduction in any medium or format, as long as you give appropriate credit to the original author(s) and the source, provide a link to the Creative Commons license, and indicate if changes were made.
In cases where reviewers are anonymous, credit should be given to 'Anonymous Referee' and the source.
The images or other third party material in this Peer Review File are included in the article's Creative Commons license, unless indicated otherwise in a credit line to the material. If material is not included in the article's Creative Commons license and your intended use is not permitted by statutory regulation or exceeds the permitted use, you will need to obtain permission directly from the copyright holder.
To view a copy of this license, visit https://creativecommons.org/licenses/by/4.0/
